# Combined Application of Orthogonal Sortases and Depsipeptide Substrates for Dual Protein Labeling

**DOI:** 10.1021/acs.bioconjchem.2c00411

**Published:** 2022-11-10

**Authors:** Holly E. Morgan, Zoe L. P. Arnott, Tomasz P. Kamiński, W. Bruce Turnbull, Michael E. Webb

**Affiliations:** School of Chemistry and Astbury Centre for Structural Molecular Biology, University of Leeds, Leeds LS2 9JT, U.K.

## Abstract

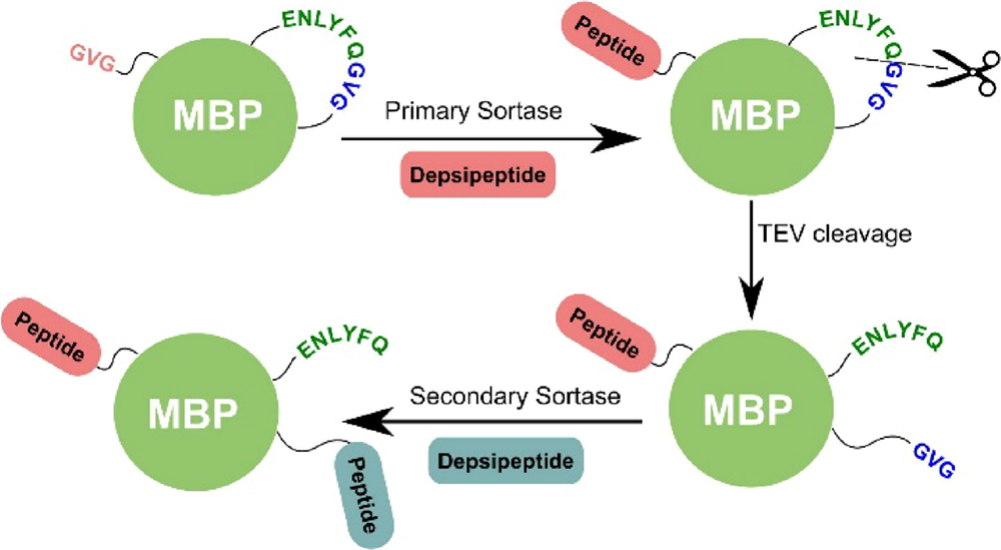

*Staphylococcus aureus* sortase A is a transpeptidase that has been extensively exploited for site-specific modification of proteins and was originally used to attach a labeling reagent containing an LPXTG recognition sequence to a protein or peptide with an N-terminal glycine. Sortase mutants with other recognition sequences have also been reported, but in all cases, the reversibility of the transpeptidation reaction limits the efficiency of sortase-mediated labeling reactions. For the wildtype sortase, depsipeptide substrates, in which the scissile peptide bond is replaced with an ester, allow effectively irreversible sortase-mediated labeling as the alcohol byproduct is a poor competing nucleophile. In this paper, the use of depsipeptide substrates for evolved sortase variants is reported. Substrate specificities of three sortases have been investigated allowing identification of an orthogonal pair of enzymes accepting LPEToG and LPESoG depsipeptides, which have been applied to dual N-terminal labeling of a model protein mutant containing a second, latent N-terminal glycine residue. The method provides an efficient orthogonal site-specific labeling technique that further expands the biochemical protein labeling toolkit.

Transpeptidase enzymes have proven to be attractive tools for protein labeling because they can allow site-specific modification at the N- or C-termini of proteins under mild conditions near physiological pH and temperature.^1,2^ Their applications range from introduction of affinity and fluorescent tags to preparation of biopharmaceuticals such as antibody–drug conjugates.^3^ Several classes of trans-peptidase have been adopted for biotechnology applications including subtilisin-derived ligases^4,5^ and peptidyl asparaginyl ligases,^6−9^ but the most popular in recent years are the sortases,^10,11^ whose natural role is to attach proteins to the cell wall of Gram-positive bacteria.^12^ In particular, *Staphylococcus aureus* sortase A (SaSrtA), which can attach a labeling reagent containing an LPXTG recognition sequence to a protein with an N-terminal glycine residue (Figure 1A).^13,14^ Several modifications have been explored to improve the efficiency of the sortase-labeling technique^15^ These include increasing the catalytic activity of the enzyme^16,17^ and addressing the enzyme’s calcium dependence.^18^ However, one of the main limitations of sortase-mediated labeling is that it is reversible (Figure 1A). The glycinyl side-product of the reaction becomes a second nucleophilic substrate for the enzyme, allowing cleavage of the label from the product. Consequently, a large excess of labeling reagent and sortase is required to push the equilibrium toward formation of labeled protein. Approaches to combat this problem include deactivation of the labeled product by forming a β-hairpin at the LPXTG site,^19^ and deactivation of the nucleophilic byproduct through formation of a diketopiperazine,^20^ or complexation with metal ions.^21^ We have previously reported the use of depsipeptide substrates, in which the amide bond between the threonine and glycine residues is replaced by an ester linkage (Figure 1B).^22−24^ The sortase reaction with depsipeptide substrates releases a poorly nucleophilic alcohol byproduct and effectively allows irreversible N-terminal labeling of proteins while using only a small excess of substrate and catalytic quantities of sortase.

**Figure 1:**
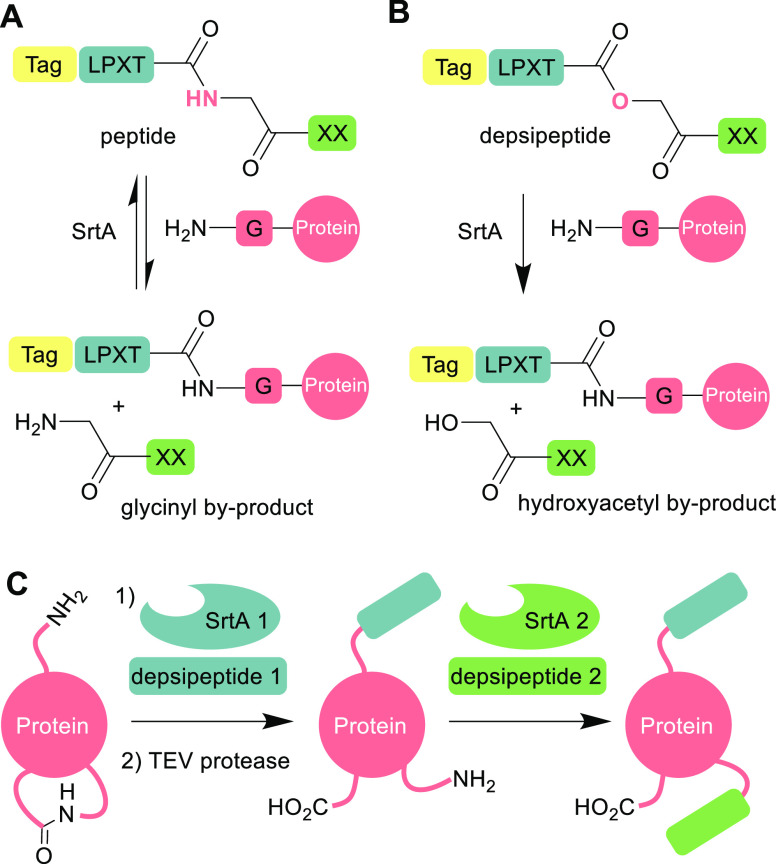
(A) Reversible labeling of a protein with a peptide tag containing an LPXTG recognition sequence for SrtA; (B) irreversible labeling of a protein with a depsipeptide tag containing an LPXToG recognition sequence for SrtA; (C) stepwise dual labeling of a protein with orthogonal SrtA enzymes and depsipeptide substrates. An intermediate TEV-protease step reveals a second latent N-terminus.

Although the substrate specificity of SaSrtA is a great advantage for site-specific protein labeling, if two different labels are to be attached to the same protein, then a second enzyme with orthogonal specificity is required. For example, *Streptococcus pyogenes* sortase A (SpSrtA), which has an LPXTA recognition sequence, has been used in conjunction with SaSrtA to label N- and C-termini of the same protein,^25^ and N- and/or C-termini of multiple proteins in the same M13 bacteriophage particle.^26,27^ Novel sortases that recognize APXTG or FPXTG sequences have been discovered through phage-display techniques,^28,29^ while eSrtA(2A-9) and eSrtA(4S-9) enzymes, identified by yeast display, have been reported to recognize LAXTG or LPXSG sequences, respectively, with high activity.^30^ Very recently, these latter two enzymes have been applied in orthogonal C-terminal labeling of a Fab’ fragment carrying an LAETGG motif on its heavy chain and LPESGG motif on its light chain.^31^ While double sortase-mediated labeling was achieved by this approach, it required a large excess of labeling reagent (50 equiv) and 75 mol % sortase, which may not always be ideal, e.g., for direct attachment of a precious reagent, such as a cytotoxic payload.

Here, we report a comparison of wildtype SaSrtA (WTSrtA) with the eSrtA(2A-9) and eSrtA(4S-9) variants for N-terminal protein labeling, used in combination with depsipeptide substrates. (For clarity when comparing substrate preferences, we will refer to eSrtA(2A-9) and eSrtA(4S-9) by their expected substrate specificities: SrtA(LAXTG) and SrtA(LPXSG), respectively.) These reactions were optimized to achieve quantitative labeling of a model protein and the initial rates of these reactions were compared to those of the variants with substrates containing recognition motifs not specific to that enzyme. This allowed two orthogonal variants, WTSrtA and SrtA(LPXSG), to be identified and used to dual label a mutant maltose-binding protein with two N-termini (Figure 1C). Orthogonal dual N-terminal labeling of this type has previously only been reported on different proteins of a bacteriophage, and in the recent work of Fottner et al.^32^ in an extension of their genetic code expansion-based approach to incorporate internal sortase-labeling sites.^33^

## Results and Discussion

**Scheme 1:**
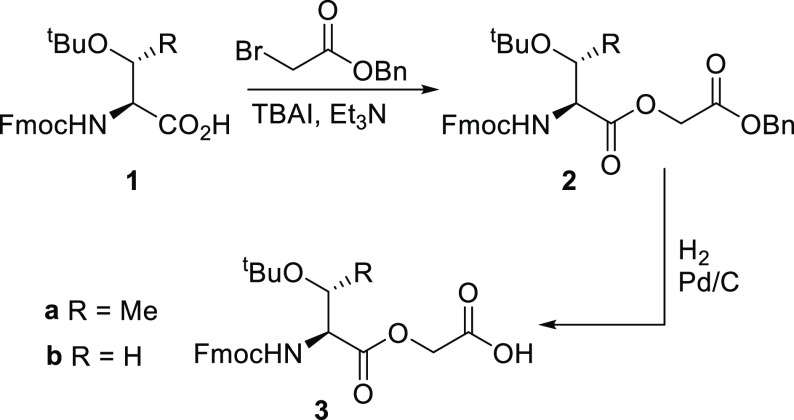
Synthesis of Protected Depsipeptide Building Blocks for Solid-Phase Peptide Synthesis

It has previously been shown that depsipeptide substrates increase the efficiency of sortase-labeling reactions with WTSrtA.^22^ To verify whether this was also true for the selected SrtA(LPXSG) and SrtA(LAXTG) variants, a model protein was labeled with analogous peptide and depsipeptide substrates. Maltose-binding protein (MBP) was chosen as the model system as it is monomeric, globular, and easy to express. It was modified with a short N-terminal GVG linker to provide GVG-MBP with an unhindered glycine residue that would be accessible for sortase labeling. The labeling substrates were designed to include the target recognition sequences of each variant enzyme (LPESG and LAETG) and a fluorescent marker for UV visualization following SDS-PAGE analysis. Standard Fmoc solid-phase peptide synthesis on 2-chlorotrityl resin was used to prepare peptide substrates (Dansyl-KALPESGG and Dansyl-KALAETGG), incorporating a dansyl group in the side chain of the N-terminal lysine residue. For depsipeptide substrates (Dansyl-KALPESoGG and Dansyl-KALAEToGG), the ester linkage replacing the scissile peptide bond was introduced using protected depsipeptide building blocks. The threonine-containing building block **3a** was prepared as previously reported^23^ and the serine-containing building block **3** was prepared in a similar fashion by sequential alkylation of Fmoc(^*t*^Bu)Ser with benzyl bromoacetate, followed by hydrogenolysis of the benzyl protecting group (Scheme 1).

**Figure 2:**
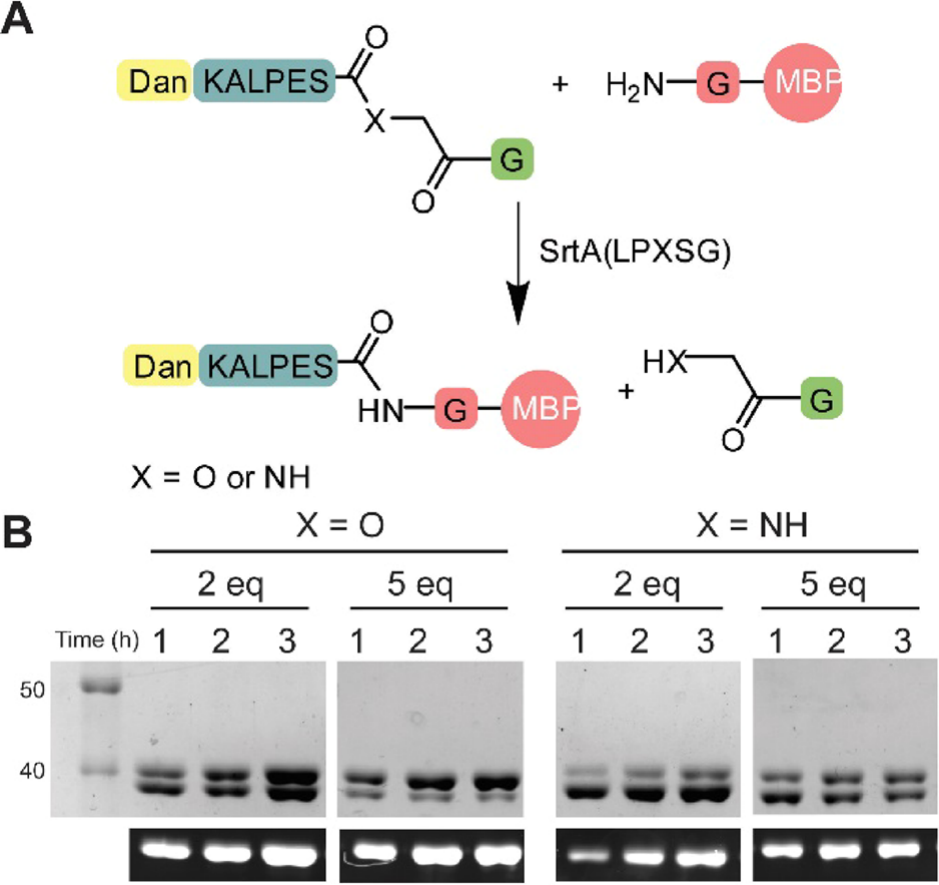
(A) General schematic of N-terminal labeling reaction using peptide and depsipeptide substates. (B) Labeling test reaction using SDS-PAGE analysis for labeling with SrtA(LPXSG) with depsipeptide and peptide reagents using 20 μM GVG-MBP, 2 μM SrtA(LPXSG) and either 40 or 100 μM of the matched peptide or depsipeptide substrate. Images are visualized with Coomassie blue stained or UV trans-illumination (only shows upper, labeled band).

Initial experiments to compare peptide and depsipeptide substrates were carried out with 20 μM GVG-MBP, 2 μM (10 mol %) sortase SrtA(LPXSG), and 2 equivalents of the fluorescently labeled depsipeptide or peptide substrate (Figure 2A) in the presence and absence of Ca^2+^ (Figure S1A). Reaction progress was monitored by SDS-PAGE followed by fluorescence imaging and Coomassie staining of the protein bands. While neither of the reactions went to completion within 4 h under these conditions, greater conversion to the product was observed for the depsipeptide substrate than for the peptide substrate, confirming that the depsipeptide substrate gave more efficient labeling as expected and the expected Ca^2+^ dependence of the reaction was observed. Subsequent optimization (Figures S2 and S3) led to optimal conditions for labeling of 20 mol % catalyst with 5 equivalents of the labeling peptide (Figure 2B). As expected, an increase in the concentration of the protein substrate also improves reaction turnover, but for the peptide substrates, even a large excess of peptide did not lead to full conversion suggesting a potential preference for the reverse reaction. For SrtA(LAXTG), complete labeling (Figure S4) was not observed with either peptide or depsipeptide substrates, and substantially larger concentrations of the catalyst were required to drive the reaction to completion, suggesting poor processivity by this enzyme.

Initial screening of SrtA(LPXSG) against the noncognate substrates suggested good orthogonality against the LAETG substrate and some reactivity with the LPETG substrates (Figure S5). For quantitative analysis of the three catalysts, the optimized conditions for labeling with SrtA(LPXSG) were used. GVG-MBP (100 μM) was incubated with each sortase (WTSrtA, SrtA(LAXTG) (20 μM) and SrtA(LPXSG) (10 μM) with concentrations selected due to the different observed specific activities) and each of the three depsipeptide substrates (5 equiv, 0.5 mM). Two different approaches were taken to reaction analysis, densitometry, and mass spectrometry. Initially, reaction timepoints were quenched in the SDS-loading buffer and analyzed by SDS-PAGE using densitometry of either UV-visualized or Coomassie-stained gels. In each case, the higher-molecular-weight band, corresponding to the fluorescently labeled product increased over time (Figure S6A). In practice, quantitation of the UV data was not reliable due to the variability in sample loading onto the gel; however, ratiometric comparison of the intensity of unlabeled and labeled MBP bands in Coomassie-stained images could be used to determine the reaction rate over time (Figures S6B and 3). As expected, the preferred depsipeptide substrates for WTSrtA and SrtA(LPXSG) were Dan-KALPEToGG and Dan-KALPESoGG, respectively. However, SrtA(LAXTG) showed greater activity with Dan-KALPEToGG than with the substrate containing its target recognition sequence (Dan-KALAEToGG). In neither case did the reaction go to completion within the time-scale of the reaction; this, together with the lack of specificity for the reported target motif, meant this enzyme was not pursued further.

**Figure 3:**
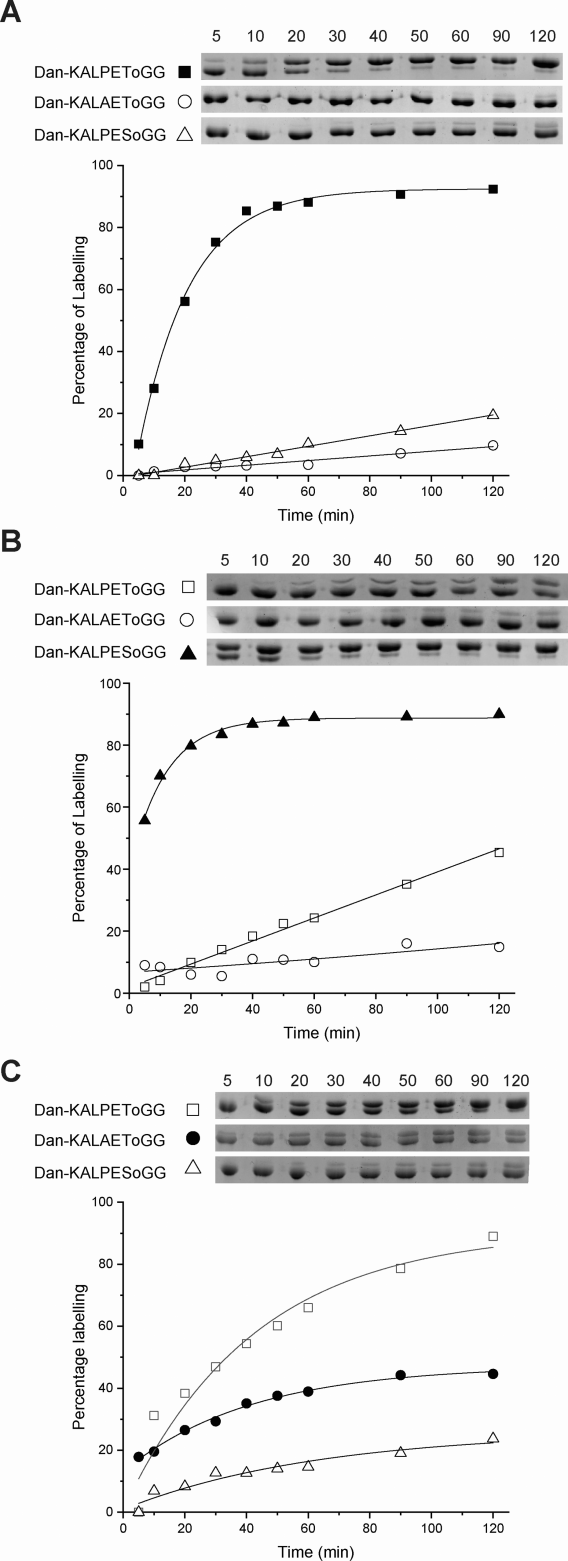
Analysis of substrate specificity of SaSrtA, SrtA(LPXSG), and SrtA(LAXTG) using depsipeptide substrates by SDS-PAGE. Reaction of 100 μM GVG-MBP with the corresponding sortase and 0.5 mM matched (filled symbol) and mismatched (open symbol) depsipeptide substrates. (A) 20 μM SaSrtA; (B) 10 μM SrtA(LPXSG); (C) 20 μM SrtA(LAXTG).

Since resolution of the labeled and unlabeled bands was not always clear by SDS-PAGE, electrospray mass spectrometry was also used to confirm the quantitation of the relative rates of the SrtA(LPXSG) and WTSrtA against the cognate and noncognate substrates. Although the presence of a small peptide tag would not prevent equivalent ionization of the labeled and unlabeled species, we anticipated that the presence of a dansyl group in the labeled protein might prevent it. New depsipeptide substrates containing recognition sequences for SaSrtA(LPXSG) and WTSrtA (AYLPESoGG and AYLPEToGG, respectively) were therefore synthesized. Each reaction mixture (100 μM GVG-MBP, 10 μM catalyst, 0.5 mM substrate) was incubated, and samples were taken at defined timepoints quenched by fivefold dilution into EGTA (final concentration 2 mM). Deconvolution of the spectra yielded a direct estimate of the degree of labeling (Figures S7 and S8). For 20 μM WTSrtA, the rate of labeling GVG-MBP was 6.3 ± 0.1 μM min^–1^ with AYLPEToGG, and 0.23 ± 0.11 μM min^–1^ with AYLPESoGG, which is 30-fold slower than the reaction with the target recognition sequence. For 10 μM SrtA(LPXSG), the rate with AYLPESoGG and AYLPEToGG substrates was 7.1 ± 1.1 and 0.17 ± 0.06 μM min^–1^, respectively; a 40-fold preference for the expected substrate sequence. (The corresponding analysis using SDS-PAGE gave rates of 5.5 ± 0.4 μM min^–1^, (WT with LPEToG), 0.16 ± 0.01 μM min^–1^ (WT with LPEToG), 7.6 ± 0.8 μM min^–1^ (SrtA(LPXSG) with LPESoG) and 0.37 ± 0.01 μM min^–1^ (SrtA(LPXSG) with LPEToG).)

To demonstrate dual labeling, a protein with two N-termini was required. The GVG-MBP construct was modified to insert a flexible loop containing a second GVG sequence that could be revealed via cleavage with TEV protease (Figure S9) between residues 177 and 178. Residue Asp177 sits at the end of a β-sheet and at the start of a natural loop in the protein, which is distant from the maltose-binding site; thus, insertion at this site was not expected to disrupt the protein’s integrity. A nucleotide sequence encoding the desired GSNSNSNSGNGGENLYFQGVG was inserted into the GVG-MBP plasmid using a Q5 site-directed mutagenesis approach. The expressed protein, MBPins, overexpressed well and could be readily purified at a yield of 28 mg/L, which was about half of that produced for GVG-MBP. We next confirmed that the protein had remained stable following TEV cleavage and did not separate into two individual peptide chains. Samples of MBPins (2 mg/mL), before and after cleavage with TEV protease, were analyzed by size-exclusion chromatography and showed the same elution profile (Figure S10).

**Figure 4:**
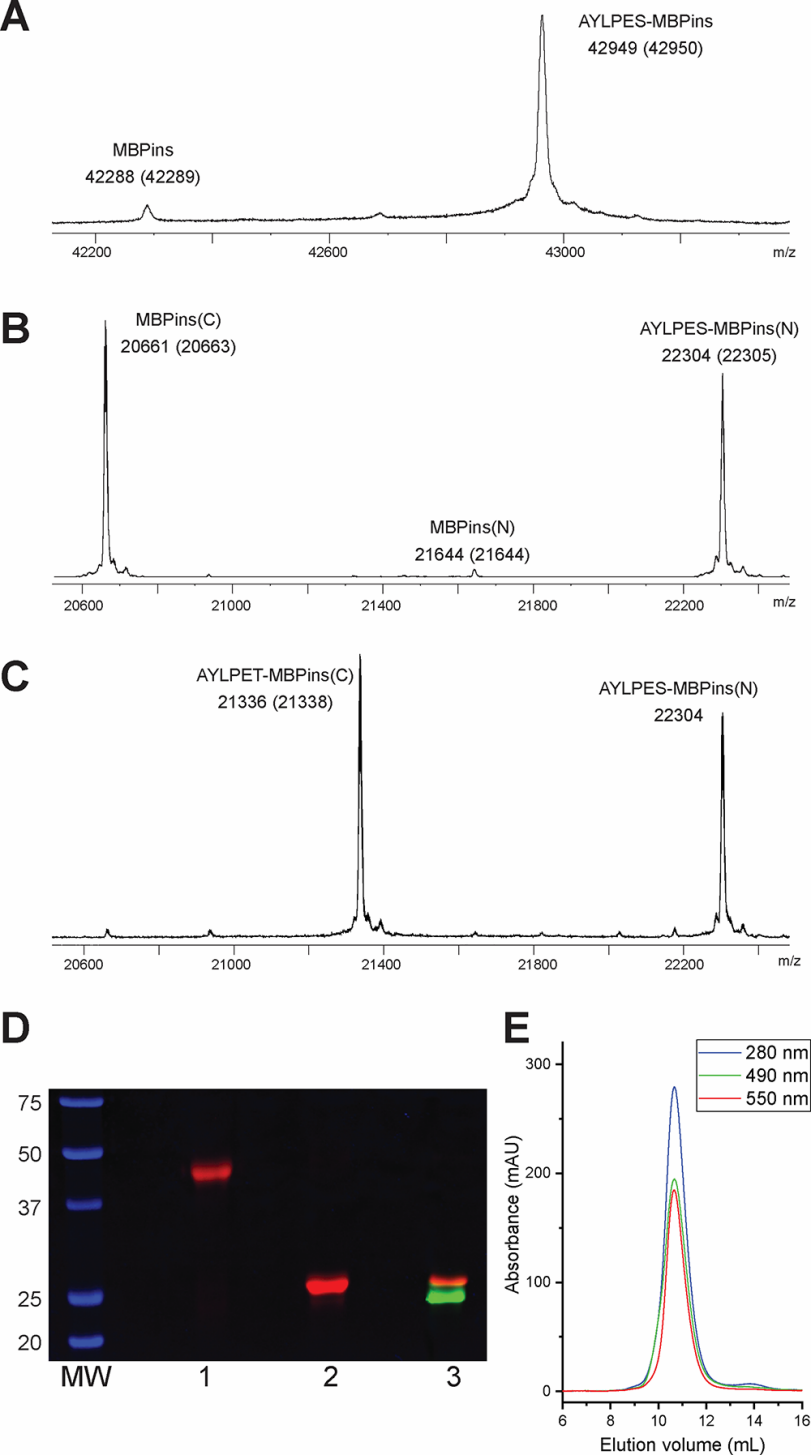
Dual labeling of a model protein at two N-termini using depsipeptide substrates A–C ESMS analysis of stepwise labeling of MBPins using SrtA(LPXSG) and WT SrtA. Expected masses are shown in parentheses. (A) MBPins labeling with SrtA(LPXSG). (B) TEV cleavage of AYLPES-MBPins to yield AYLPES-MBPins(N) and MPBins(C). (C) MBPins(C) labeling with WTSrtA. (D) Dual labeling of MBPins with fluorescent depspipeptides (1—reaction of TAMRA-LPESoG depsipeptide with MBPins; 2—TEV cleavage of TAMRA-MBPins; 3—reaction of Fluor-LPEToG depsipeptide with MBPins(C)). (E) SEC analysis of dual labeling MBPins indicates the protein tertiary structure is retained. All masses are within 1 Da of expected peak mass.

The strategy for orthogonal dual labeling of MBPins is outlined in Figure 1C. The N-terminus was first labeled with SrtA(LPXSG)—MBPins (100 μM) was treated with 20 mol % SrtA(LPXSG) and 5 equiv of AYLPESoGG. Reaction progress was monitored via mass spectrometry, and near-quantitative labeling was achieved after 2.5 h (Figure 4A), at which point the reaction was quenched by addition of EGTA followed by SrtA(LPXSG) catalyst removal using Ni-NTA affinity chromatography. TEV site cleavage of AYLPES-MBPins to yield AYLPES-MBPins(N) and MBPins(C) was performed with 20 mol % TEV-H_6_ (Figure 4B). The peak at 20,661 Da corresponds to the C-terminal portion of the protein (MBPins(C)). The peaks at 22,304 and 21,644 Da correspond to the labeled and residual N-terminal portion of the mutant, respectively. The protease was removed by nickel affinity chromatography and diafiltration in a centrifugal concentrator was used to remove EGTA and remaining excess depsipeptide from the cleaved AYLPES-MBPins protein (final concentration 40 μM). Quantitative labeling of the revealed secondary site was achieved in 1 h, 20 mol % WTSrtA, and 5 equiv AYLPEToGG (Figure 4C). A mass shift of the peak corresponding to the C-terminal portion of the mutant can be observed, with the new peak at 21,336 Da being consistent with addition of AYLPET. It is imperative that the sortase variants are orthogonal for the dual labeling to work, otherwise the secondary sortase would remove the label from the primary labeling site and labeling with the secondary enzyme could then occur at both sites. No removal of label or cross-reactivity was seen at either labeling site. Dual labeling of MBPins with these peptide substrates was also achieved with the sortases applied in the opposite order, i.e., with WTSrtA acting on the N-terminus of MBPins and SrtA(LPXSG) acting on the N-terminus revealed by TEV cleavage (Figure S11).

Dual labeling of GVG-MBP(loopinsert) was also performed with distinct fluorescent markers to demonstrate that functional peptides could be attached to the protein and to allow further analysis to be carried out. SrtA(LPXSG) acted as the primary sortase variant, this time using TAMRA-GABA-AVLEAYLPESoGG as a substrate (Figure S12A). Following TEV cleavage, secondary labeling was performed with WTSrtA and fluorescein-GABA-YLPEToGG (Figure S12B and S12C). The final product was purified via size-exclusion chromatography. Analysis by SDS-PAGE at each step of the labeling procedure demonstrated successful orthogonal labeling of the protein (Figure 4D). Fluorescence imaging using different excitation wavelengths for the fluorescein and TAMRA groups demonstrated that each polypeptide chain was labeled with a distinct fluorophore. Size-exclusion chromatography of the dual labeled MBP mutant on a Superdex 75 Increase 10/300 GL column was monitored at three wavelengths corresponding to the natural protein absorbance (280 nm), as well as the UV absorption of fluorescein (490 nm) and TAMRA (550 nm). The traces showed that the labeled protein elutes at the same volume as the intact protein (Figures 4E and S10) prior to labeling, again indicating that the protein remains the same size and TEV cleavage does not lead to dissociation of the two polypeptide chains. We further confirmed the maltose-binding properties of the labeled protein by confirming that the labeled protein still interacted with amylose resin in a maltose-dependent fashion (Figure S14).

## Conclusions

In this study, the application of depsipeptide substrates in conjunction with sortase A variants, WTSrtA, SrtA(LPXSG), and SrtA(LAXTG), has been investigated. Previously, the use of depsipeptide substrates for improving the efficiency of N-terminal labeling has only been demonstrated with WTSrtA.^22^ Here, we have shown that reactions with a sortase A variant with altered specificity (SrtA(LPXSG)) can also be improved with depsipeptide substrates. The specificity of the three sortase A variants with depsipeptide substrates was also explored. Variants reprogrammed to accept different recognition sequences are not necessarily specific to using that sequence. For example, SrtA(LAXTG) shows acceptance of its target recognition sequence; however, it has a preference for the LPETG recognition sequence. SrtA(LPXSG), on the other hand, does show a preference for the LPXSG sequence. WTSrtA and SrtA(LPXSG) are suitable candidates for orthogonal labeling of proteins as they both show specificity to their target sequences and little promiscuity toward the opposing sequence.

Orthogonal dual labeling has been reported previously with two sortases from different species at the N- and C-termini of a protein.^25^ It has also been carried out at two C-termini of a multimeric protein with two variant enzymes based on SrtA mutants from the same species.^31^ In this study, we have demonstrated orthogonal dual labeling on a protein engineered to have two N-termini. The sortases selected, WTSrtA and SrtA(LPXSG), could be used interchangeably for the first and second labeling reactions and to incorporate two distinct fluorescent markers at specific sites in the MBP mutant using this technique. Fluorescently labeled analyte-binding proteins of this type have potential for the development of molecular sensors that detect structural change in the protein upon binding via FRET though a preliminary analysis of the labeled protein indicate that such a change is not observed for the pair of sites selected in this study. More broadly, we anticipate that the depsipeptide substrate approach that we have developed will be readily adaptable to other dual N-terminal labeling approaches such as that of Fottner et al.^20^ and to, for example, the labeling of distinct N-termini in heteromeric protein complexes and effectively increases the range of applications for this class of transpeptidase substrates.

## Experimental Procedures

### Fmoc-Ser(O*t*Bu)-Gc-OBn 2-(benzyloxy)-2-oxoethyl *N*-(((9*H*-fluoren-9-yl)methoxy)carbonyl)-*O*-(*tert*-butyl)serinate **2b**

Fmoc-Ser(O*t*Bu)-OH **1b** (2 g, 5.22 mmol) was dissolved in THF (10 mL). Benzyl 2-bromoacetate (1.24 mL, 7.83 mmol), tert-butylammonium iodide (0.77 g, 2.09 mmol) and triethylamine (0.87 mL, 6.26 mmol) were added sequentially and the reaction was stirred overnight at room temperature. The reaction mixture was washed with H_2_O (200 mL) and the crude product extracted with ethyl acetate (2 × 150 mL). The ethyl acetate layers were combined and washed with sodium thiosulfate solution (10% w/v) (2 × 300 mL) and sodium chloride solution (40% w/v) (300 mL). The ethyl acetate layers were combined, dried with sodium sulfate, and concentrated to yield a yellow oil. The title compound was obtained via flash column chromatography 4:1 (v/v) hexane/EtOAc as a colorless powder (2.4 g, 43%). *R*_F_: 0.43 (2:1 (v/v) hexane/EtOAc); ^1^H NMR (400 MHz (CD_3_OD): δ 7.80 (2H, d, *J* = 7.6 Hz), 7.67 (2H, dd, *J* = 7.7, 3.3 Hz), 7.39, 7.41–7.28 (9H, m), 5.19 (2H, s), 4.75 (2H, d, *J* = 2.4 Hz) 4.49 (1H, t, *J* = 4.6 Hz), 4.39–4.31 (2H, m), 4.24 (1H, dt, *J* = 7.0, 3.5 Hz, *J*_H7–H2_ 3.5 Hz), 3.77 (1H, dd, *J* = 9.2, 5.3 Hz) 3.69 (1H, dd, *J* = 9.2, 4.0 Hz), 1.17 (9H, s) ^13^C NMR (100 MHz, CD_3_OD): 170.2, 167.5, 157.0, 143.9, 141.2, 135.5, 128.2, 128.0, 127.9, 127.4, 126.8, 124.9, 119.5, 73.4, 66.8, 66.6, 61.5, 61.0, 54.9, 47.0, 26.2; IR (ν_max_/cm^–1^): 3414.1, 2962.9, 2938.5, 2886.4, 1752.90, 1724.01.

### Fmoc-Ser(O*t*Bu)-Gc-OH 2-((*N*-(((9*H*-fluoren-9-yl)methoxy)carbonyl)-*O*-(*tert*-butyl)seryl)oxy)acetic acid **3b**

Fmoc-Ser(OtBu)-Gc-OBn (1 g, 1.88 mmol) **2b** was dissolved in 6 mL of methanol/5 mL of DCM before H_2_O (4 mL) was slowly added. To the stirred solution, Pd/C (10%) (100 mg) was added and the reaction mixture was stirred under a H_2_ atmosphere for 1 h 45 min. The reaction mixture was filtered through Celite and washed with methanol (∼200 mL). The crude product was concentrated, freeze-dried to remove excess water, and purified via flash column chromatography (9:1 (v/v) CH_2_Cl_2_/EtOAc, 1% AcOH) to yield a foamy colorless solid (690 mg, 83%). *R*_F_: 0.10 (9:1 (v/v) CH_2_Cl_2_/EtOAc, 1% AcOH); ^1^H NMR (400 MHz, CDCl_3_): 7.76 (2H, d, *J* = 7.5 Hz), 7.61 (2H, app t, *J* = 6.7 Hz), 7.40 (2H, t, *J* = 7.4 Hz), 7.31 (2H, t, *J* = 7.4 Hz), 5.71 (1H, d, *J* = 8.5 Hz), 4.79 (1H, d, *J* = 16.3 Hz), 4.70 (1H, d, *J* = 16.3 Hz), 4.60 (1H, dt, *J* = 8.5, 3.2 Hz), 4.44 (1H, dd, *J* = 10.6, 7.4 Hz), 4.39 (1H, dd, *J* = 10.6, 7.2 Hz), 4.25 (1H, app t, *J* = 7.1 Hz), 3.91 (1H, dd, *J* = 9.2, 3.1 Hz), 3.67 (1H, dd, *J* = 9.2, 3.2 Hz), 1.17 (9H, s); ^13^C-NMR (100 MHz, CD_3_OD): 170.2, 169.6, 157.0, 143.8, 141.2, 127.4, 126.8, 124.9, 119.5, 73.3, 66.8, 61.5, 61.0, 55.0, 47.0, 26.3; IR (*ν*_max_/cm^–1^): 3423.1, 2973.6, 2859.5, 1764.45, 1725.5; HRMS (ES): found [M + Na]^+^ 464.1680, C_24_H_27_NO_7_Na requires 464.1680.

## Supporting Information Available

General procedures for reaction analysis. All experimental procedures pertaining to the production and characterization of proteins and substrates. ^1^H, ^13^C NMR and HPLC analytical data for substrates (PDF)

## Notes

The data associated with this paper are openly available from the University of Leeds data repository: https://doi.org/10.5518/1258.
