# Supplementary material for: Combined Application of Orthogonal Sortases and Depsipeptide Substrates for Dual Protein Labeling
Source: Bioconjug Chem. 2022 Nov 10;33(12):2341–7. doi: 10.1021/acs.bioconjchem.2c00411 (PMC9782347; doi:10.1021/acs.bioconjchem.2c00411)
Supplement: Supplementary file 1 — bc2c00411_si_001.pdf [file bc2c00411_si_001.pdf]

Supporting Information for

Combined application of orthogonal sortases and depsipeptide substrates for dual protein labelling

Holly E. Morgan, Zoe L. P. Arnott, Tomasz P. Kamiński, W. Bruce Turnbull* and Michael E. Webb*

School of Chemistry and Astbury Centre for Structural Molecular Biology, University of Leeds, Leeds LS2 9JT, UK

*Corresponding authors: w.b.turnbull@leeds.ac.uk, m.e.webb@leeds.ac.uk

Figure S1. Initial screening of Srt(LPXSG) activity as a function of Ca^{2+} and enzyme concentration. **A** 20 μM GVG-MBP incubated with 2 μM Srt(LPXSG) and 40 μM Dan-KALPETоGG depsipeptide or Dan-KALPETGG peptide substrate (2 equivalents) in the presence and absence of Ca^{2+}. **B** 20 μM GVG-MBP incubated with 1μM Srt(LPXSG) and 40 μM Dan-KALPETоGG or Dan-KALPETGG depsipeptide or peptide substrate **C.** 20 μM GVG-MBP incubated with 2 μM Srt(LPXSG) and 40 μM Dan-KALPETоGG or Dan-KALPETGG substrate

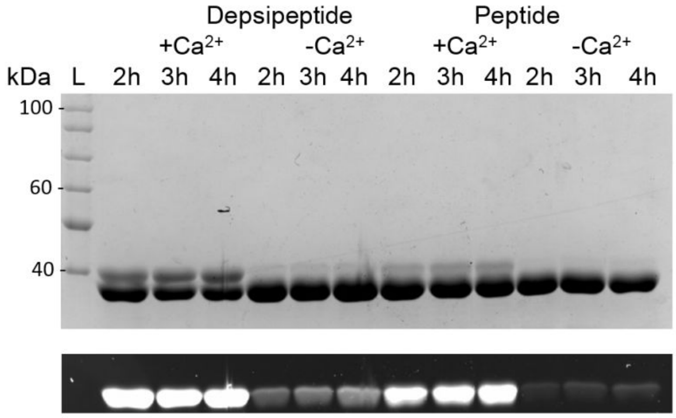

A

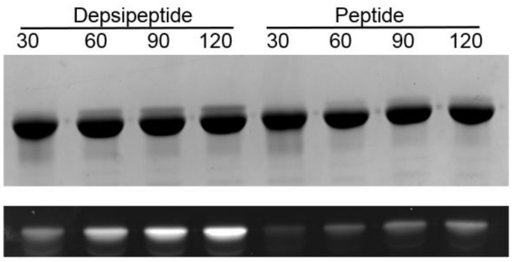

B

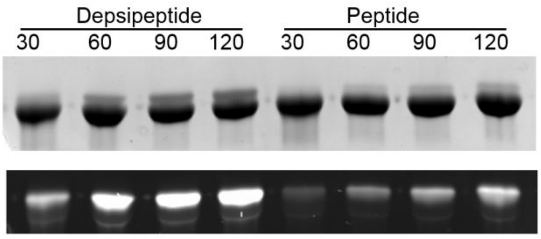

C

Figure S2. Variation in Srt(LPXSG) labelling level with peptide/depsipeptide substrate concentration. **A&B** Incubation of 20 μM GVG-MBP with 4 μM Srt(LPXSG) and 40 μM (2 eq), 100 μM (5 eq) or 200 μM (10 eq) Dan-KALPESGG or Dan-KALPESoGG substrate **C**. Incubation of 20 μM GVG-MBP with 100 μM peptide/depsipeptide substrate and 4 μM (20%) and 10 μM (50%) SrtA(LPXSG)

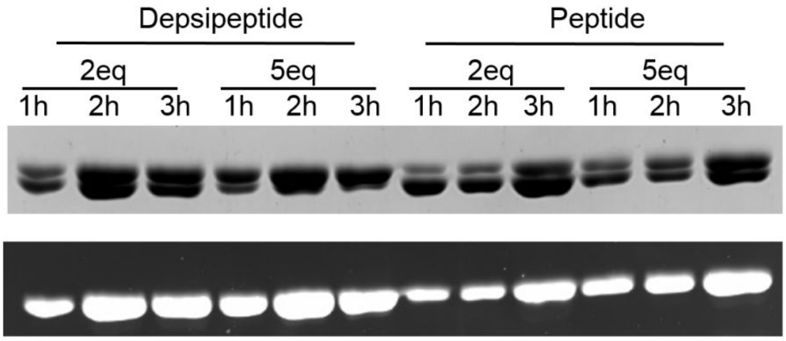

A

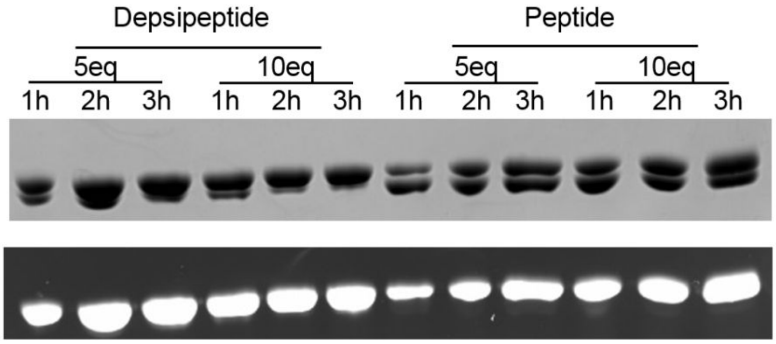

B

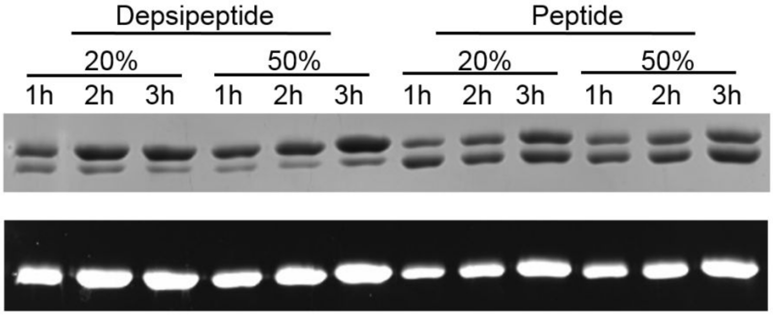

C

Figure S3 Variation in SrtA(LPXSG) labelling with variation in GVG-MBP concentration **A**. Incubation of 20 μM or 50 μM GVG-MBP with 4 μM SrtA(LPXSG) and 100 μM peptide/depsipeptide substrate, and 10 μM SrtA(LPXSG) and 250 μM peptide/depsipeptide respectively. B. Incubation of 150 μM GVG-MBP with 30 μM SrtA(LPXSG) and 750 μM depsipeptide substrate and incubation of 100 μM GVG-MBP with 20 μM SrtA(LPXSG) and either 500 μM or 350 μM depsipeptide substrate.

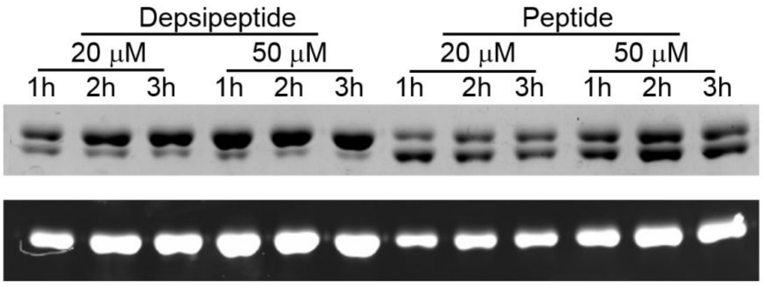

A

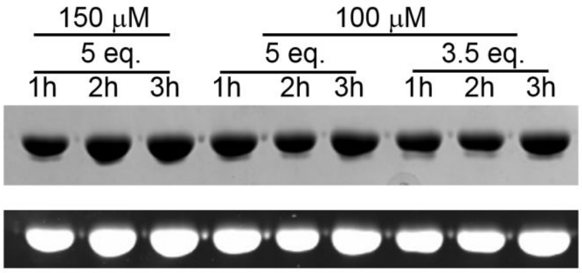

B

Figure S4 Screening of WT SrtA and Srt(LAXTG) activity at optimal labelling conditions for SrtA(LPXSG) **A**. Incubation of 100 μM GVG-MBP with 20 μM SrtA and 500 μM Dan-KALPEToGG depsipeptide substrate. **B.** Incubation of 100 μM GVG-MBP with 20 μM SrtA(LAXTG) and 500 μM Dan-KALAEToGG depsipeptide. **C.** Incubation of 100 μM GVG-MBP with 500 μM Dan-KALAEToGG and 20 μM SrtA(LAXTG). Additional aliquots of SrtA(LAXTG) added at 60 and 120 min (final concentration 60 μM)

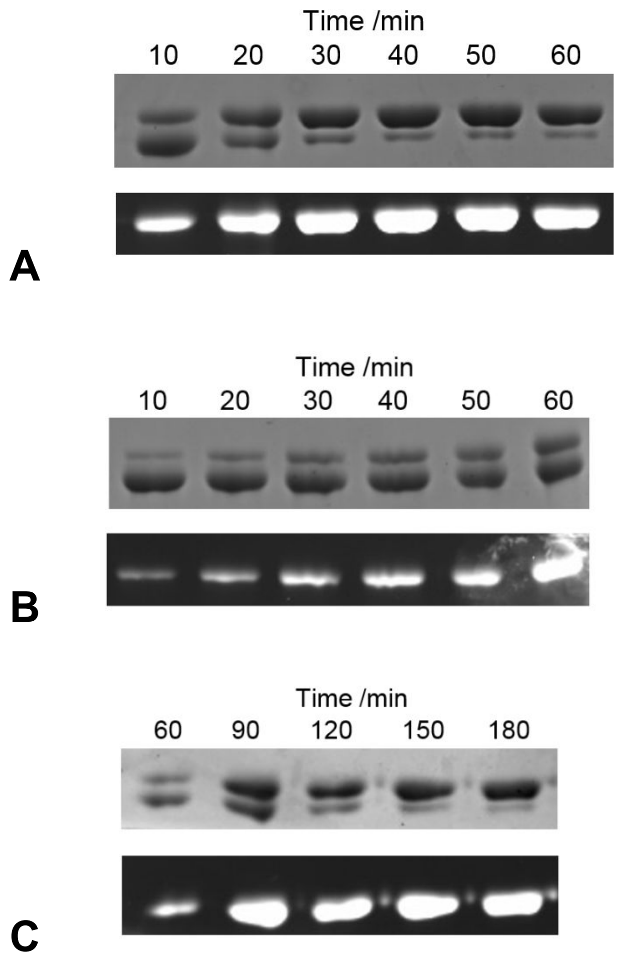

Figure S5 Screening of activity of SrtA(LPXSG) against cognate and non-cognate substrate. Incubation of 100 μM GVG-MBP with 10 μM SrtA(LPXSG) and 500 μM of the indicated depsipeptide substrate.

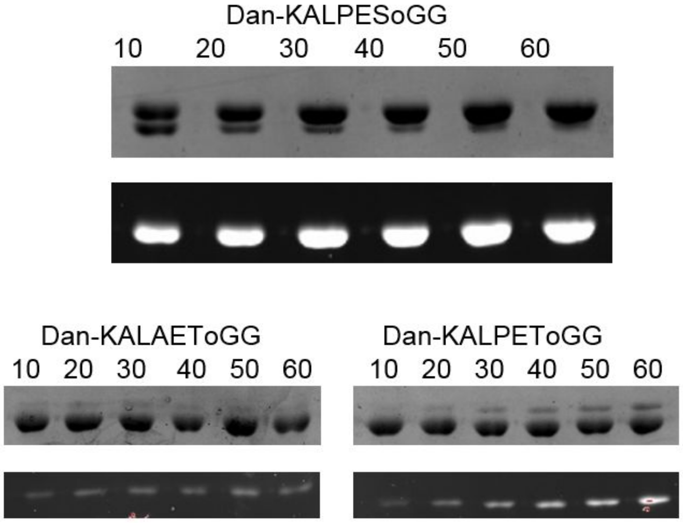

Figure S6. Comparison of two different methods of data analysis for the same experiment. 100 μM GVG-MBP was incubated with 20 μM WTSrtA and 500 μM Dan-KALPETоGG. After separation by SDS-PAGE, the protein labelling was quantified using both UV-induced fluorescence (**A**) and ratiometric quantification of the labelled and unlabelled species using Coomassie-staining (**B**). Greater random scatter is observed in the fluorescence labelling approach.

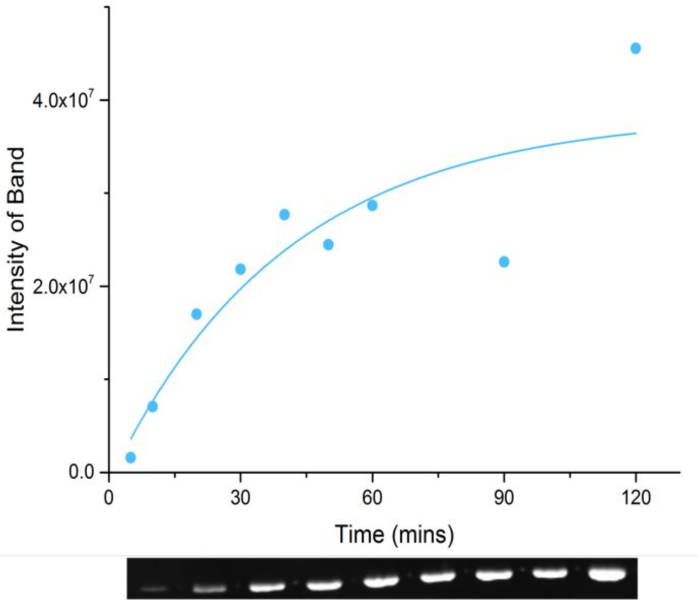

A

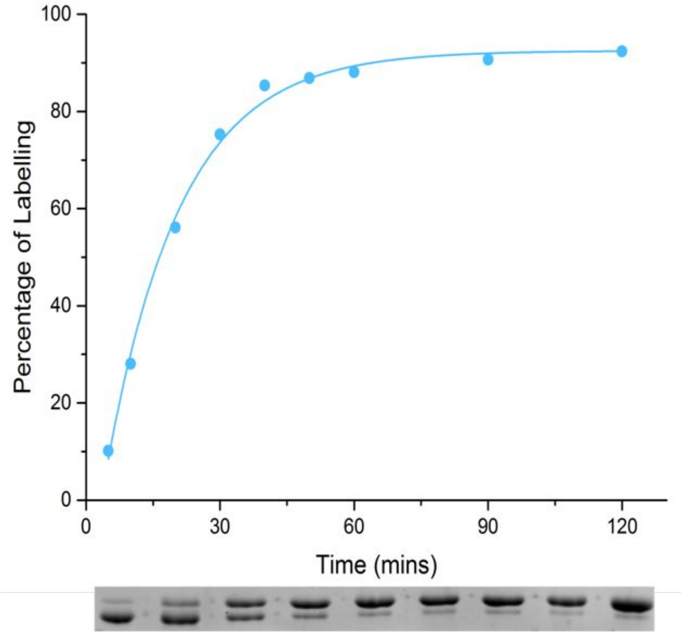

B

Figure S7 Exemplar data obtained for ESMS based-analysis of GVG-MBP labelling. Individual samples were diluted into 100% MeCN and analysed by LC-MS. Spectra corresponding to GVG-MBP (2.2-2.3 min) were combined and deconvolved to yield a quantitative estimate of reaction progression.

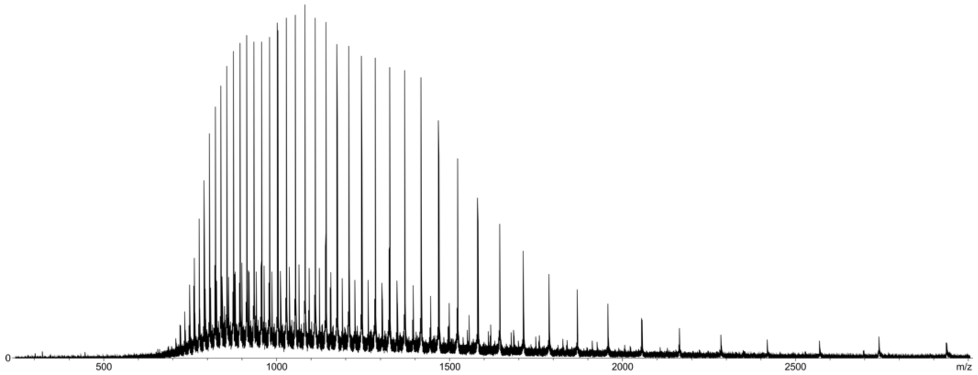

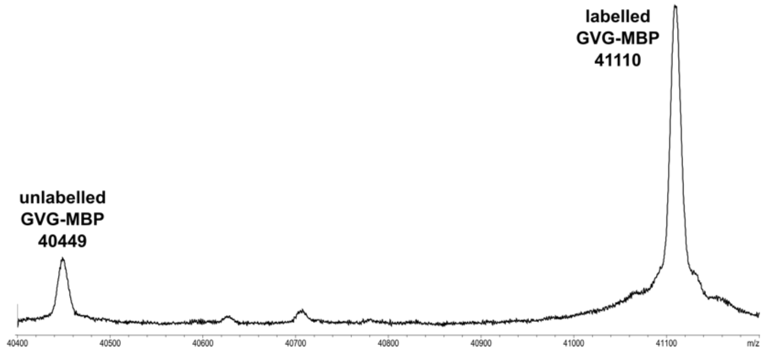

Figure S8. Time course of labelling of GVG-MBP using WT SrtA and SrtA(LPXSG) with cognate and non-cognate depsipeptide substrates. 100 μM GVG-MBP was incubated either 10 μM SrtA(LPXSG) or 20 μM WT SrtA and either 500 μM YALPEToGG (squares) or YALPESoGG substrates (triangles). Filled symbols indicate the cognate substrate. Samples were analysed by LC-MS as shown in supplementary figure 7. **A** Reaction of SrtA(LPXSG) **B** Reaction of WT SrtA.

A s

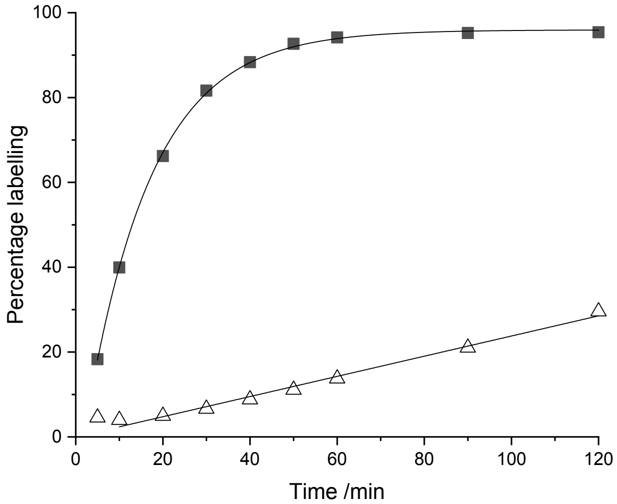

B

Figure S9 Location of loop insertion sequence in maltose-binding protein A X-ray crystal structure of wild type MBP (PDB: 1ANF) with Asp177, the site of insertion, highlighted in yellow. **B** Alignment of protein sequence of wild type MBP and the MBP mutant containing an N-terminal GVG (purple) and loop insert with a spacer (red), TEV cleavage site (green) and second GVG (blue).

A

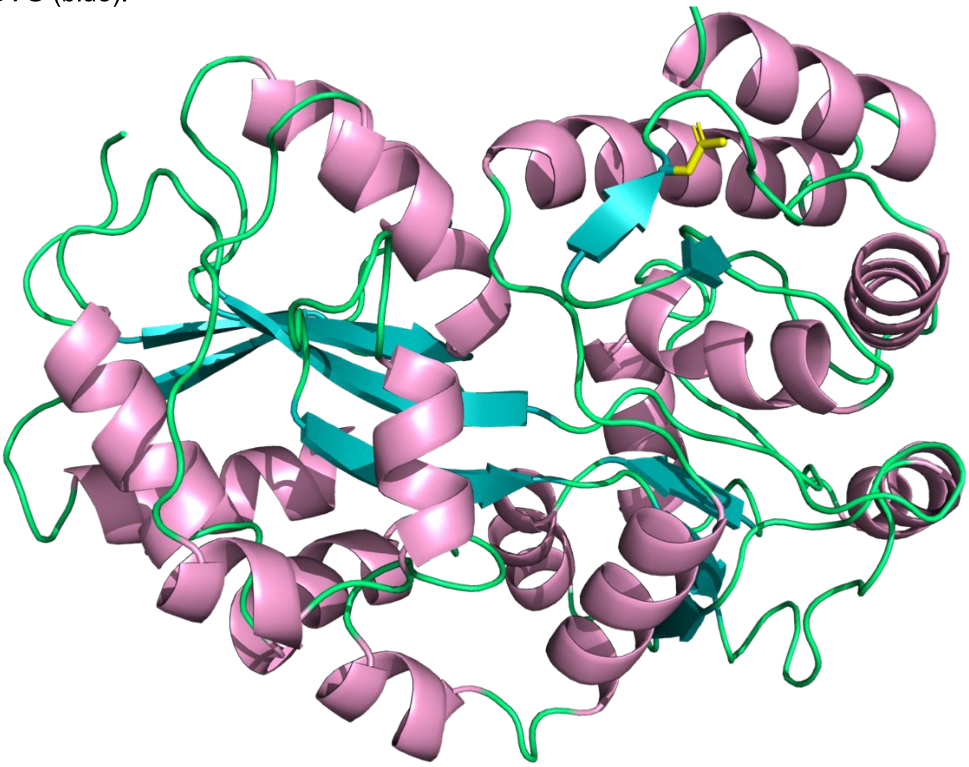

B

```
WTMBP       - - - K I E E G K L V I W I N G D K G Y N G L A E V G K K F E K D T G I K V T V
MBPmutant   G V G . . . . . . . . . . . . . . . . . . . . . . . . . . . . . . . . . . . . .

WTMBP       E H P D K L E E K F P Q V A A T G D G P D I I F W A H D R F G G Y A Q S G L L A
MBPmutant   . . . . . . . . . . . . . . . . . . . . . . . . . . . . . . . . . . . . . . . .

WTMBP       E I T P D K A F Q D K L Y P F T W D A V R Y N G K L I A Y P I A V E A L S L I Y
MBPmutant   . . . . . . . . . . . . . . . . . . . . . . . . . . . . . . . . . . . . . . . .

WTMBP       N K D L L P N P P K T W E E I P A L D K E L K A K G K S A L M F N L Q E P Y F T
MBPmutant   . . . . . . . . . . . . . . . . . . . . . . . . . . . . . . . . . . . . . . . .
                                                                                     Asp177
WTMBP       W P L I A A D G G Y A F K Y E N G K Y - - - - - - - - - - - - - - - - - - - D I
MBPmutant   . . . . . . . . . . . . . . . . . . . G S N S N S G N G G E N L Y F Q G V G . .

WTMBP       K D V G V D N A G A K A G L T F L V D L I K N K H M N A D T D Y S I A E A A F N
MBPmutant   . . . . . . . . . . . . . . . . . . . . . . . . . . . . . . . . . . . . . . . .

WTMBP       K G E T A M T I N G P W A W S N I D T S K V N Y G V T V L P T F K G Q P S K P F
MBPmutant   . . . . . . . . . . . . . . . . . . . . . . . . . . . . . . . . . . . . . . . .

WTMBP       V G V L S A G I N A A S P N K E L A K E F L E N Y L L T D E G L E A V N K D K P
MBPmutant   . . . . . . . . . . . . . . . . . . . . . . . . . . . . . . . . . . . . . . . .

WTMBP       L G A V A L K S Y E E E L A K D P R I A A T M E N A Q K G E I M P N I P Q M S A
MBPmutant   . . . . . . . . . . . . . V . . . . . . . . . . . . . . . . . . . . . . . . . .

WTMBP       F W Y A V R T A V I N A A S G R Q T V D E A L K D A Q T R I T K
MBPmutant   . . . . . . . . . . . . . . . . . . . . . . . . . . . . - - - -
```

Figure S10: Size-exclusion chromatography analysis of MBP(ins) before and after treatment with HisTEV, and after double labelling. Chromatogram of MBP(ins) (green) and MBP(ins) after treatment with His-TEV (red) resulting from isocratic elution through a Superose 75 increase (10/300) column.

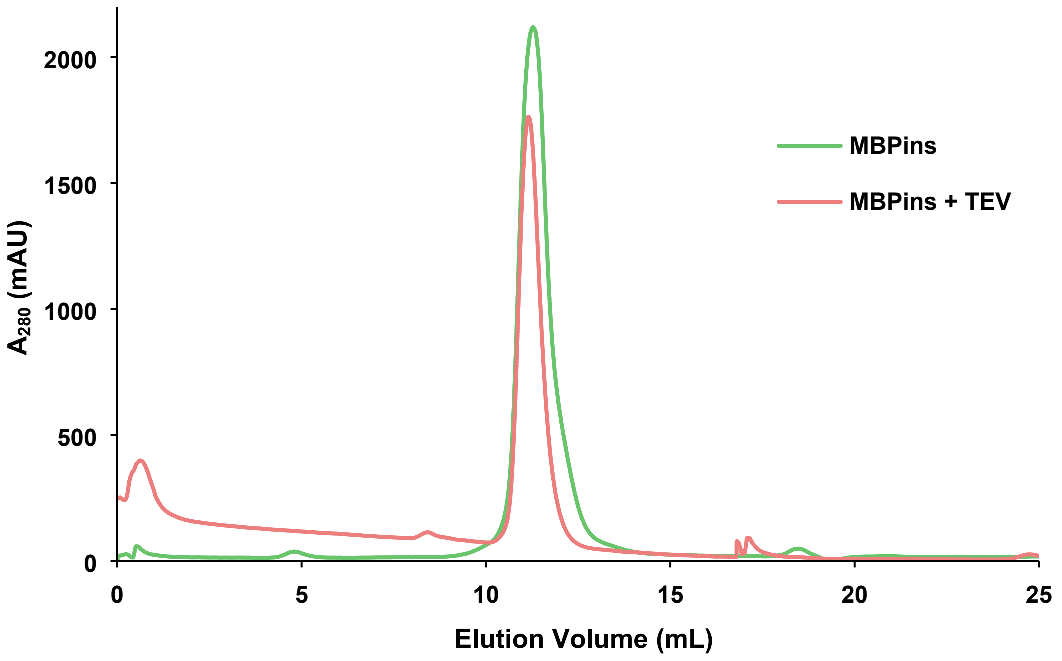

Figure S11: Mass spectrometry data confirming the successful labelling of both N-termini of MBP mutant with WTSrtA and SrtA(LPXSG) acting as the primary and secondary variant respectively. A. Deconvoluted MS trace of GVG-MBP(loopinsert) before labelling. B. Primary sortase labelling with WTSrtA, C. TEV cleavage results in the mutant appearing as two separate peaks in the trace. The ‘N-term-degraded’ corresponds to the labelled N-terminal in which the SNSGNGGENLYFQ portion of the inserted loop has been lost following trace proteolysis. D. Secondary sortase labelling with SrtA(LPXSG).

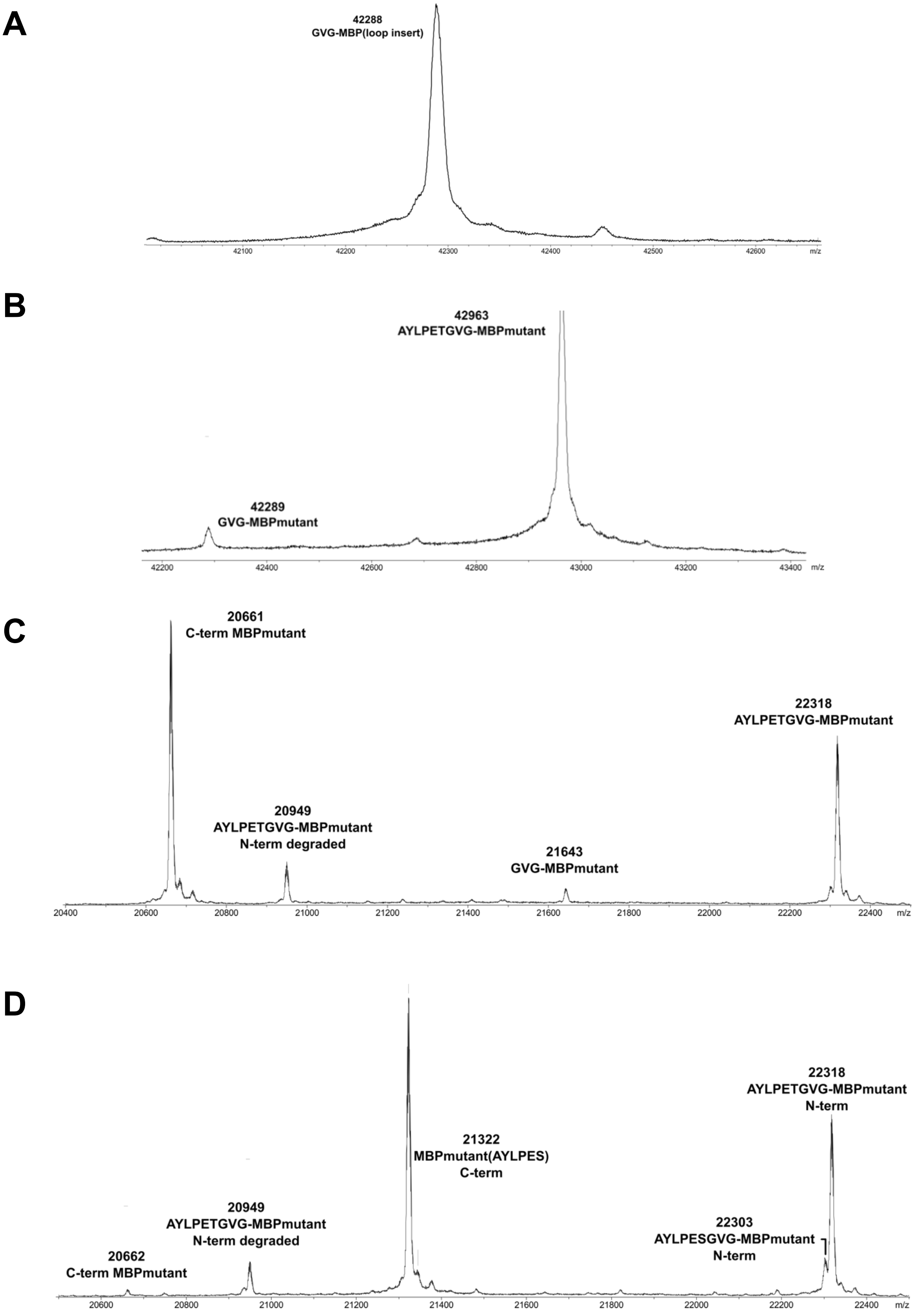

Figure S12: Mass spectrometry data confirming the successful labelling of both N-termini of MBP mutant with SrtA(LPXSG) and WTSrtA acting as the primary and secondary variant respectively with fluorescent depsipeptides. A. Primary sortase-labelling with SrtA(LPXSG) using a TAMRA-depsipeptide. Note the presence of contaminating peaks due to impurities in the TAMRA depsipeptide. B. TEV cleavage, C. Secondary sortase labelling with WTSrtA.

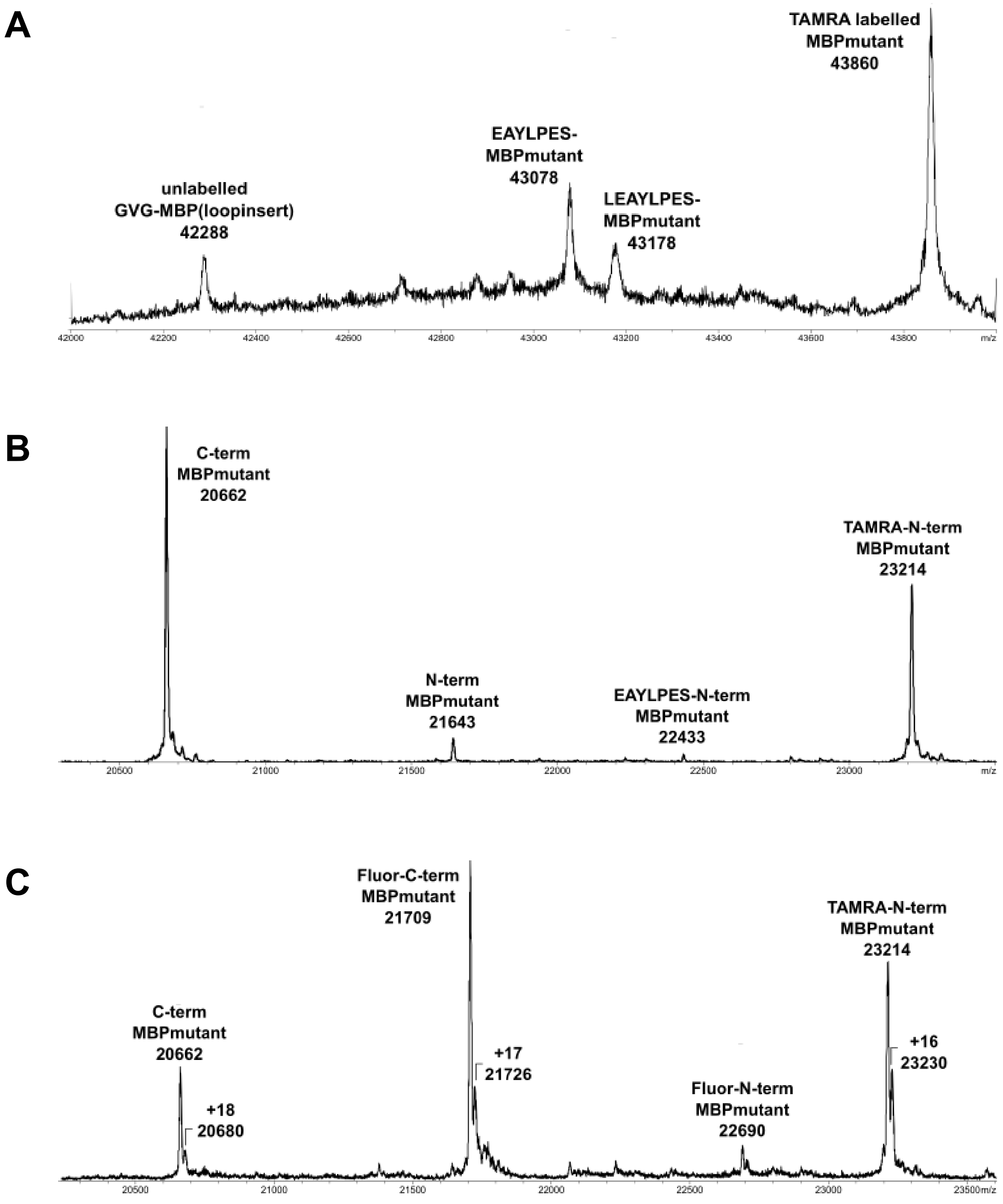

Figure S13: 15% resolving SDS-PAGE gel of fluorescent dual labelled MBP. Samples were taken after 1) primary labelling – TAMRA-labelled MBP, 2) TEV cleavage – TAMRA labelled N-terminal fragment (top), unlabelled C-terminal fragment, 3) secondary labelling with fluorescein peptide – TAMRA labelled N-terminal fragment and fluorescein-labelled C-terminal fragment, 4) protein after size exclusion chromatography demonstrating co-elution of the two labelled protein fragments. The gel was imaged using UV-induced fluorescence of A TAMRA (550 nm) and B fluorescein (490 nm) and C after Coomassie staining. *Note the primary sequence-dependence of Coomassie staining reflected by the different intensity of the protein bands for each part of MBPins despite their equimolar concentrations.*

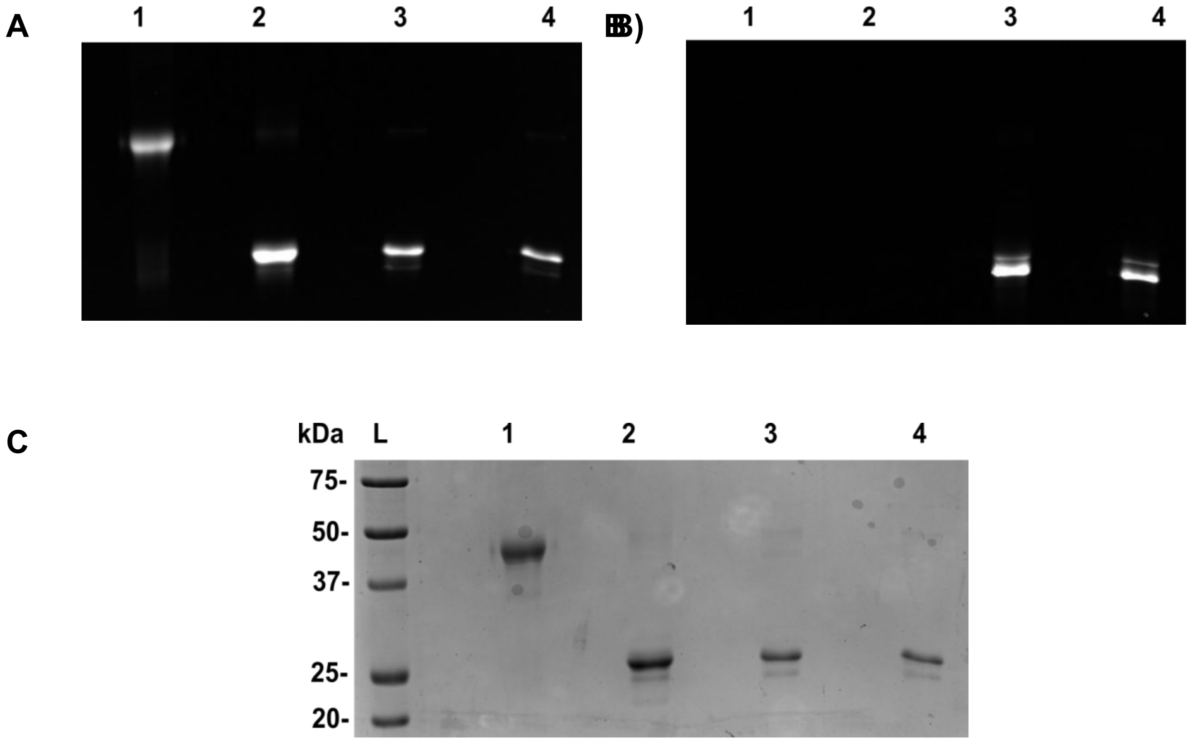

Figure S14 Binding of doubly-fluorescent labelled MBPins to amylose resin A) Fluorescently dual-labelled MBP mutant eluted from the size exclusion column was collected and concentrated. It appears as a pinky-orange colour in solution due to the combination of the pink TAMRA and yellow fluorescein groups. B) Amylose resin was added to the solution and the mixture was applied to a spin filter. Upon centrifugation, the resin remained in the top of the filter and the solution flowed through. C) 10 mM maltose buffer was added to the top of the spin filter, releasing the protein from the resin and allowing it to flow through in the solution.

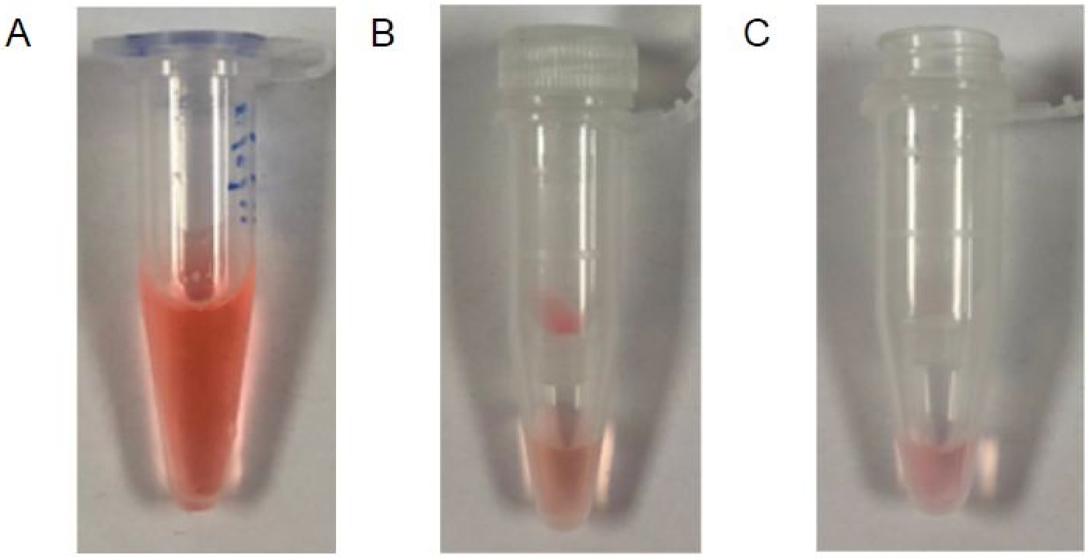

General protein labelling procedures.

SDS-PAGE procedure. For a total volume of 40 μL, GVG-MBP (100 μM) and labelling reagent (500 μM, 5 eq.) were mixed and made up to volume with 50 mM Tris, 150 mM NaCl pH 7.6 containing calcium chloride (reaction concentration of 1 mM) before the addition of the sortase enzyme (10 μM, 10 mol% or 20 μM, 20 mol%). The reaction mixture was incubated for 120 minutes at 37 ^{o}C and time point samples were taken (4 μL), diluted to 20 μL and mixed with SDS-PAGE loading buffer (20 μL) to dilute the GVG-MBP protein in the sample to 10 μM. The samples were boiled at 95 ^{o}C for 10 minutes and 2 μL of each sample were loaded onto a 10% acrylamide gel. The gel was developed until GVGK-MBP approached the bottom of the gel to ensure good separation between labelled and unlabelled protein (180 V, 60 min). The gel was then imaged using a what in UV transillumination (exposed for 3 minutes) and then stained with Coomassie Blue and imaged again using white light transilluimnation. The images were processed using BioRad ImageLab software to quantify the intensity of gel bands for the labelled and unlabelled species. Data were fitted using either a single phase exponential or linear fitting procedure in Origin.

Mass spectrometry procedure

GVG-MBP (100 μM) and depsipeptide substrate (500 μM, 5 eq.) were mixed and made up to volume with 50 mM Tris, 150 mM NaCl pH 7.6 containing 1 mM $CaCl_2$ before the addition of the sortase variant (10 μM, 10 mol%). The reaction mixture was incubated at 37 °C for 2hr. Time point samples were taken from each reaction (2 μL), diluted to 20 μM with water and 2 mM EGTA was added to the sample to quench the reaction. The samples were analysed via high resolution mass spectrometry on a Bruker Daltonics MicroTOF mass spectrometer. The time region where GVG-MBP is present (2.1-2.3 minutes) was deconvoluted to give a trace with unlabelled and labelled peaks (Individual samples were diluted into 100% MeCN and analysed by LC-MS. Spectra corresponding to GVG-MBP (2.2-2.3 min) were combined and deconvolved). The peaks were quantified on the basis of peak height. Data were fitted using either a single phase exponential or linear fitting procedure in Origin.

Protocols for dual labelling of GV-MBP(ins)

Primary labelling with SrtA(LPXSG) and secondary labelling with WTSrtA

AYLPESoGG (500 μM, 5 eq.) was mixed with GVG-MBP(loopinsert) (100 μM) in 50 mM Tris, 150 mM NaCl pH 7.6 containing 1 mM $CaCl_2$. To the reaction was added SrtA(LPXSG) (20 μM, 20 mol%) for a total volume of 250 μL. The reaction mixture was incubated at 37 °C for 2 hr and then quenched with EGTA (2 mM). The reaction mixture was applied to a short nickel column (~ 3mL amylose resin) and eluted with EGTA buffer to remove excess sortase. TEV cleavage (20 mol% TEV-H_6, RT, 1 hr) was perfomed to reveal the secondary labelling site and after completion, excess protease was removed via nickel column chromatography. The eluant was concentrated and washed with lysis buffer to remove EGTA and excess depsipeptide in a spin filter (Amicon 10 kDa MWCO). Secondary labelling of MBP (40 μM) was performed by adding AYLPEToGG (200 μM, 5 eq.) in lysis buffer containing 1 mM $CaCl_2$. WTSrtA (8 μM, 20 mol%) was added for a total volume of 20 μL and the reaction mixture was incubated at 37 °C for 1 hr.

Primary labelling with WTSrtA and secondary labelling with SrtA(LPXSG)

AYLPEToGG (500 μM, 5 eq.) was mixed with GVG-MBP(loopinsert) (100 μM) in 50 mM Tris, 150 mM NaCl pH 7.6 containing 1 mM $CaCl_2$. To the reaction was added WTSrtA (20 μM, 20 mol%) for a total volume of 500 μL. The reaction mixture was incubated at 37 °C for 2.5 hr (90% labelling achieved). The reaction was quenched with EGTA (2mM). The reaction mixture was applied to a short amylose column (~ 3mL amylose resin) and washed with EGTA buffer to remove sortase and labelled MBP was eluted with 10 mM maltose in EGTA buffer. The eluant was concentrated and washed with lysis buffer to remove EGTA and excess depsipeptide in a spin filter (Amicon 10 kDa MWCO). TEV cleavage (20 mol% TEV-H_6, RT) was performed to reveal the secondary labelling site, with monitoring by MS) (A further peak at 20949 was present in this trace. This corresponds to the labelled N-terminal portion of the mutant, with a portion of the inserted loop missing. It can be assumed that this occurs because, after TEV cleavage, the portion of the loop containing the TEV site becomes flexible and susceptible to degradation. Once the TEV cleavage reaction had gone to

completion, excess protease was removed with nickel resin. Secondary labelling of MBP (40 μM) was performed by adding AYLPESoGG (200 μM, 5 eq.) in 50 mM Tris, 150 mM NaCl pH 7.6 containing 1 mM $CaCl_2$. SrtA(LPXSG) (8 μM, 20 mol%) was added for a total volume of 20 μL and the reaction mixture was incubated at 37 °C for 2.5 hr.

Labelling with fluorescent markers

TAMRA-GABA-AVLEAYLPESoGG (500 μM, 5 eq.) was mixed with GVG-MBP(loopinsert) (100 μM) in PBS buffer containing 1 mM $CaCl_2$. To the reaction was added SrtA(LPXSG) (20 μM, 20 mol%) for a total volume of 500 μL. The reaction mixture was incubated at 37 °C for 2.5 hr and applied to a short nickel column (~ 3mL nickel resin) to remove excess sortase and washed with PBS buffer. The eluant and wash fractions were concentrated and washed with PBS to remove excess depsipeptide via diafiltration in a spin filter (Amicon 10 kDa MWCO). The concentration of the primary labelled protein was determined using an extinction coefficient of 69330 ($M^{-1}cm^{-1}$). TEV cleavage was performed by mixing primary labelled MBP (100 μM) with TEV-H_6 (20 μM, 20 mol%) and shaking for 2 hours at RT. Excess protease was removed via nickel chromatography and the eluant was concentrated via diafiltration. Secondary labelling of MBP (100 μM) was performed by adding Fluorescein-GABA-AYLPEToGG (500 μM, 5 eq.) in PBS buffer containing 1 mM $CaCl_2$. WTSrtA (20 μM, 20 mol%) was added and the reaction mixture was incubated at 37 °C for 2.5 hr. Nickel chromatography and diafiltration were performed to remove sortase and excess depsipeptide. The reaction mixture was further purified via size exclusion chromatography (Superdex 75 Increase 10/300 GL) .The reaction mixture was made up to 500 μL with PBS buffer and injected onto the column and eluted using PBS buffer with a flow rate of 0.8 mL/min. Protein elution was monitored via 280, 490 and 550 nm UV absorption. The fractions containing protein were concentrated using a spin filter (Amicon 10 kDa MWCO). Each step of the reaction was analysed via HRMS and SDS-PAGE.

Preparation of expression constructs

Sortases The expression construct for WT SrtA was used as described previously. Expression plasmids of SrtA(LAXTG) and SrtA(LPXSG) were obtained by gene synthesis. Briefly, the coding sequences for eSrtA(2A-9) (SrtA(LAXTG)) and eSrtA(4S-9) (SrtA(LPXSG)), including mutations identified by Dorr *et al.* in the SaSrtAΔN59 sequences. Sequences including a 5'-UTR sequence including a rbs sequence and no stop codon with flanking NheI and XhoI sites in the pTwist32 CHLOR Uni9 vector (Twist Biosciences) were were subcloned into the Xba1/XhoI sites of pET28a using NheI/XhoI to generate expression constructs for the C-terminally hexahis-tagged proteins. (Sequences below).

```
SrtA(LPXSG)
T/CTAGGAATAATTTTGTTTAACTTTAAGAAGGAGATATACCATGCAAGCTAAACCTCAAATTCCGAAAG
ATAAATCAAAAGTGGCAGGCTATATTGAAATTCCAGATGCTGATATTAAAGAACCAGTATATCCAGGACC
AGCAACACCTGAACAATTAGATAGAGGTGTATGCTTTGTAGAAGAAAATGAATCACTAGATGATCAAAAT
ATTTCAATTACAGGACACACTGCCATTGACCGTCCGAACTATCAATTTACAAATCTTAGAGCAGCCAAAA
AAGGTAGTATGGTGTACTTAAAAGTTGGTAATGAAACACGTAAGTATAAAATGACAAGTATAAGAGACGT
TAAGCCAACAGATGTAGAAGTTCTAGATGAACAAAAAGGTAAAGATAAACAATTAACATTAgTTACTTGT
GATGATTACAATTTTAAGACAGGCGTTTGGGAAAAACGTAAAATCTTTGTAGCTACAGAAGTCAAACTCG
AGCACCACCACCACCACCACTGA

MQAKPQIPKDKSKVAGYIEIPDADIKEPVYPGPATPEQLDRGVCFVEENESLDDQNISITGHTAIDRPNY
QFTNLRAAKKGSMVYLKVGNETRKYKMTSIRDVKPTDVEVLDEQKGKDKQLTLVTCDDYNFKTGVWEKRK
IFVATEVKLEHHHHHH*

SrtA(LAXTG)
T/CTAGGAATAATTTTGTTTAACTTTAAGAAGGAGATATACCATGCAAGCTAAACCTCAAATTCCGAAAG
ATAAATCAAAAGTGGCAGGCTATATTGAAATTCCAGATGCTGATATTAAAGAACCAGTATATCCAGGACC
AGCAACACCTGAACAATTAGATAGAGGTGTATGCTTTGTAGAAGAAAATGAATCACTAGATGATCAAAAT
ATTTCAATTACAGGACACACTGCCATTGACCGTCCGAACTATCAATTTACAAATCTTAGAGCAGCCAAAA
AAGGTAGTATGGTGTACTTAAAAGTTGGTAATGAAACACGTAAGTATAAAATGACAAGTATAAGAGACGT
TAAGCCAACAGATGTAGAAGTTCTAGATGAACAAAAAGGTAAAGATAAACAATTAACATTAFTTACTTGT
GATGATTACAATTTTAAGACAGGCGTTTGGGAAAAACGTAAAATCTTTGTAGCTACAGAAGTCAAACTCG
AGCACCACCACCACCACCACTGA

MQAKPQIPKDKSKVAGYIEIPDADIKEPVYPGPATPEQLNRGVCFQDENESLDDQNISIAGHTFIDRPNY
QFTNLKAAKPGSMVYFKVGNETRIYKMTSIRKVHPNDVEVLDEQEGKDKQLTLVTCDDYNEKTGVWESRK
IFVATEVKLEHHHHHH*
```

GVG-MBP The expression vector for GVG-MBP was generated via sequential Quikchange site-directed mutagenesis of the pMALc5x plasmid (New England Biolabs), to insert the coding sequence for GVG after the N-terminal start codon and a stop codon to the C-terminus of the MalE-coding sequence using primers as described below.

GVGins-F CCAACAAGGACCATAGATTATGGGCGTGGGTAAAATCGAAGAAGGTAAACTGG
GVGins-R CCAGTTTACCTTCTTCGATTTTACCCACGCCCATAATCTATGGTCCTTGTTGG
MalEStopF CTGAAAGACGCGCAGACTTAATCGAGCTCGAACAACAAC
MalEStopR GTTGTTGTTCGAGCTCGATTAAGTCTGCGCGTCTTTCAG

```
MGVGKIEEGKLVIWINGDKGYNGLAEVGKKFEKDTGIKVTVEHPDKLEEKFPQVAATGDGPDIIFWAHDR
FGGYAQSGLLAEITPDKAFQDKLYPFTWDAVRYNGKLIAYPIAVEALSLIYNKDLLPNPPKTWEEIPALD
KELKAKGKSALMFNLQEPYFTWPLIAADGGYAFKYENGKYDIKDVGVDNAGAKAGLTFLVDLIKNKHMNA
DTDYSIAEAAFNKGETAMTINGPWAWSNIDTSKVNYGVTVLPTFKGQPSKPFVGVLSAGINAASPNKELA
```

KEFLENYLLTDEGLEAVNKDKPLGAVALKSYEEELVKDPRIAATMENAQKGEIMPNIPQMSAFWYAVRTA
VINAASGRQTVDEALKDAQT*

GVG-MBP(ins) Insertional mutagenesis was performed to insert the coding sequence GSNSNSNSGNGGENLYFQGVG loop before residue Asp177 into the GVG-MBP expression construct. The Q5 procedure was used with the primers shown below to exponentially amplify a blunt-ended PCR product which was phosphorylated and ligated to generate the new expression construct.

LoopinsF CGAAAACCTGTACTTTCAGGGTGTCGGTGACATTAAAGACGTGGGC
LoopinsR CCGCCATTTCCAGAATTTGAGTTACTTCCGTACTTGCCGTTTTCATAC

MGVGKIEEGKLVIWINGDKGYNGLAEVGKKFEKDTGIKVTVEHPDKLEEKFPQVAATGDGPDIIFWAHDR
FGGYAQSGLLAEITPDKAFQDKLYPFTWDAVRYNGKLIAYPIAVEALSLIYNKDLLPNPPKTWEEIPALD
KELKAKGKSALMFNLQEPYFTWPLIAADGGYAFKYENGKYGSNSNSGNGGENLYFQGVGDIKDVGVDNAG
AKAGLTFLVDLIKNKHMNADTDYSIAEAAFNKGETAMTINGPWAWSNIDTSKVNYGVTVLPTFKGQPSKP
FVGVLSAGINAASPNKELAKEFLENYLLTDEGLEAVNKDKPLGAVALKSYEEELVKDPRIAATMENAQKG
EIMPNIPQMSAFWYAVRTAVINAASGRQTVDEALKDAQT

Production of proteins

Sortases SaSrtA(LPXSG) and SaSrtA(LAXTG), along with WTSrtA (N-terminal his-tagged) were overexpressed in *E. coli* BL21 (DE3) cells via autoinduction. The cells were incubated at 30 °C for 5 hours prior to overnight incubation at 25 °C. Isolated cell pellets were resuspended in 50 mM Tris, 150 mM NaCl pH 7.6, mechanically lysed and purified via sequential Ni-NTA affinity chromatography and size-exclusion chromatography using a Superdex® 75 column and isocratically eluted in 50 mM Tris, 150 mM NaCl pH 7.6 to yield pure protein. (see figures S15,S16,S17)

GVG-MBP was overexpressed in *E. coli* BL21 via autoinduction as described for the sortases. The cells were resuspended in 50 mM Tris, 150 mM NaCl pH 7.6, mechanically lysed and purified via sequential amylose affinity chromatography (eluted with 10 mM maltose 50 mM Tris, 150 mM NaCl pH 7.6) and size-exclusion chromatography using a Superdex® 75 column and isocratically eluted in 50 mM Tris, 150 mM NaCl pH 7.6 to yield pure protein (58 mg/L). See figure S18.

GVG-MBP(ins) protein was overexpressed in *E. coli* BL21 via autoinduction and purified in the same way as GVG-MBP to yield pure protein (28 mg/L). The data supporting successful purification of GVG-MBP(loopinsert) can be found in Figure S19.

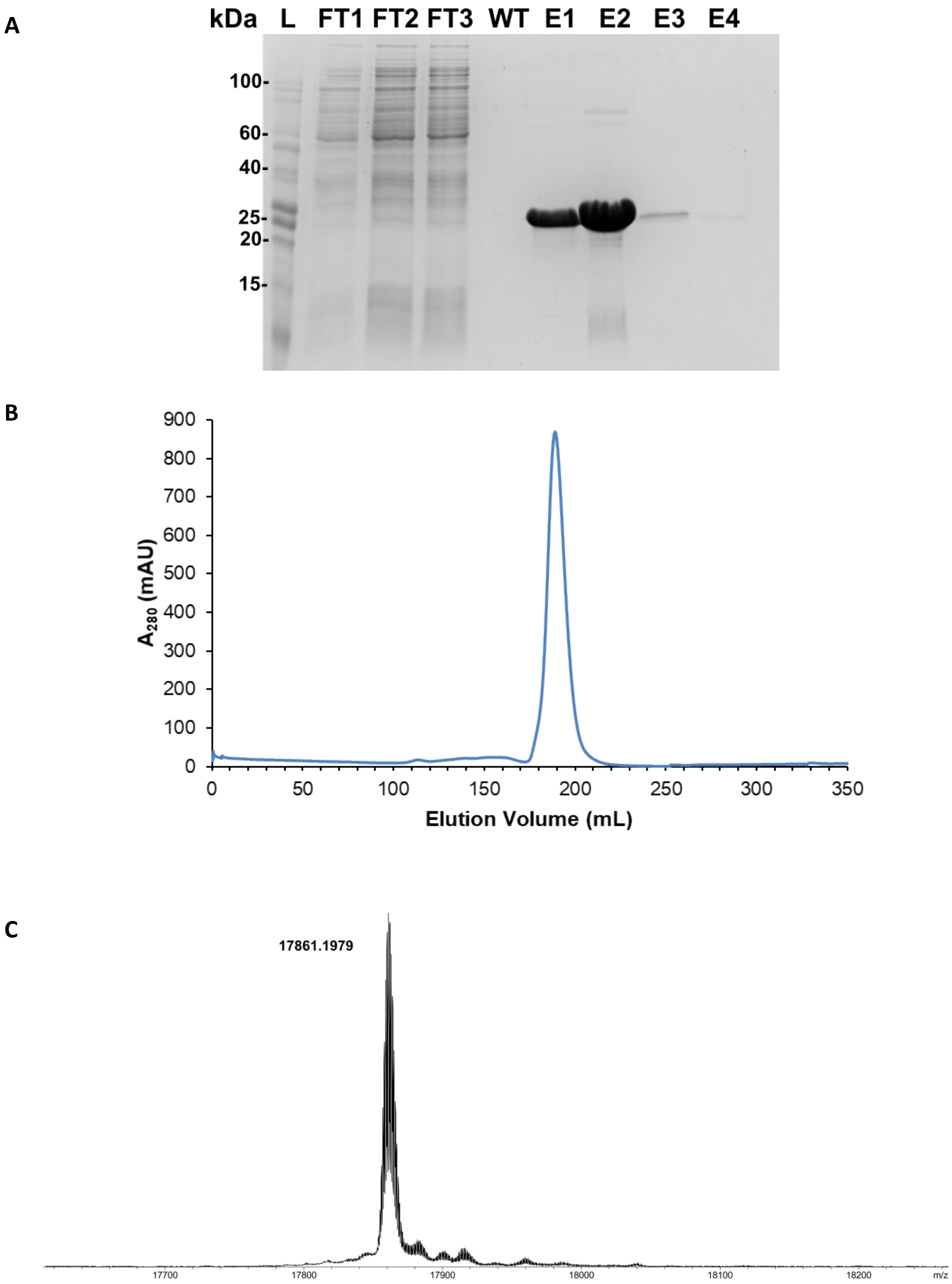

Figure S15: SDS-PAGE gel of nickel affinity purification of SrtA(LPXSG), ran alongside a protein ladder (NEB protein standard). WT = wash through, E = elution fraction. B) Chromatogram trace of elution from SEC column. Fractions containing elution volume 170-210 mL were collected and concentrated C) HRMS of fractions showed presence of SrtA(LPXSG). Expected average mass 17861 Da

A

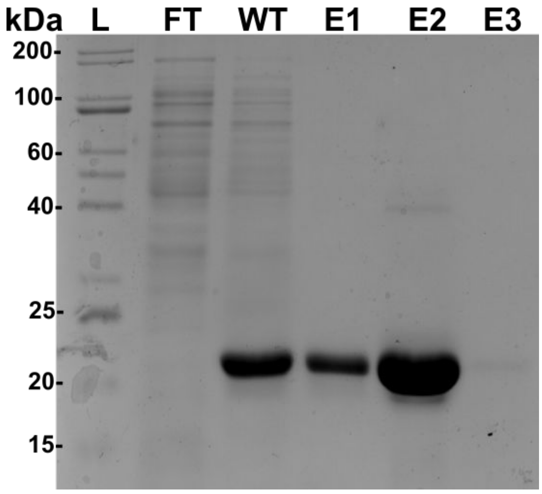
kDa
L
FT
WT
E1
E2
E3
200
100
60
40
25
20
15

B

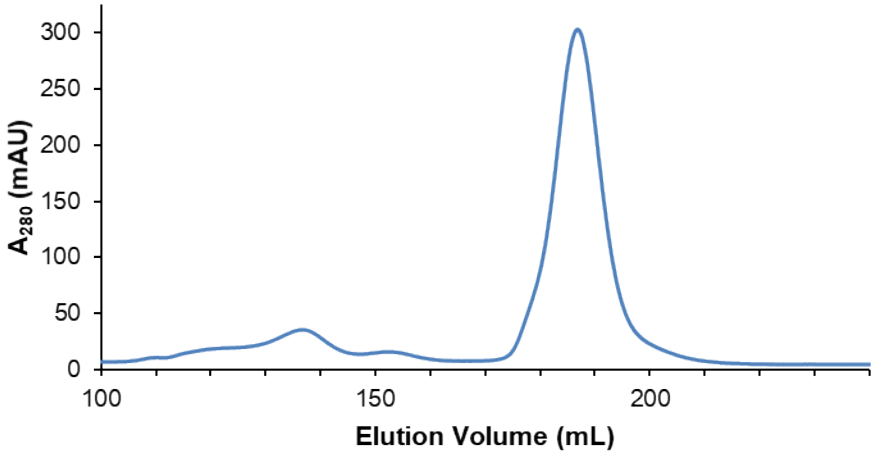
A280 (mAU)
300
250
200
150
100
50
0
100
150
200
Elution Volume (mL)

C

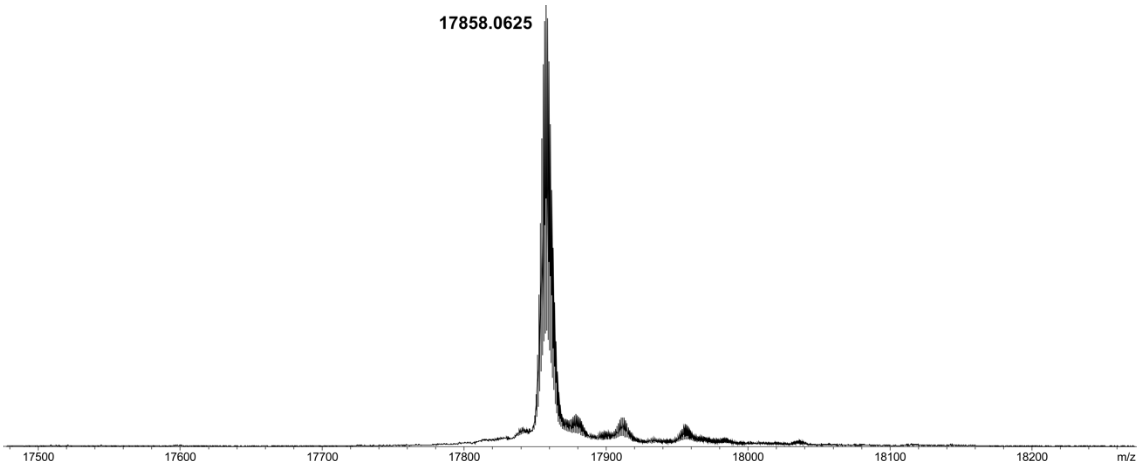
17858.0625
17500
17600
17700
17800
17900
18000
18100
18200
m/z

Figure S16: SDS-PAGE gel of nickel affinity purification of SrtA(LAXTG), ran alongside a protein ladder (NEB protein standard). WT = wash through, E = elution fraction. B) Chromatogram trace of elution from SEC column. Fractions containing elution volume 170-210 mL were collected and concentrated C) HRMS of fractions showed presence of SrtA(LAXTG). Expected average mass 17857 Da

A

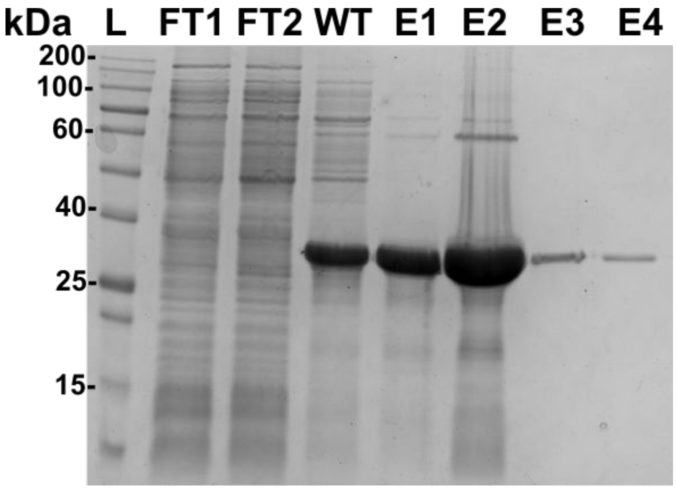

B

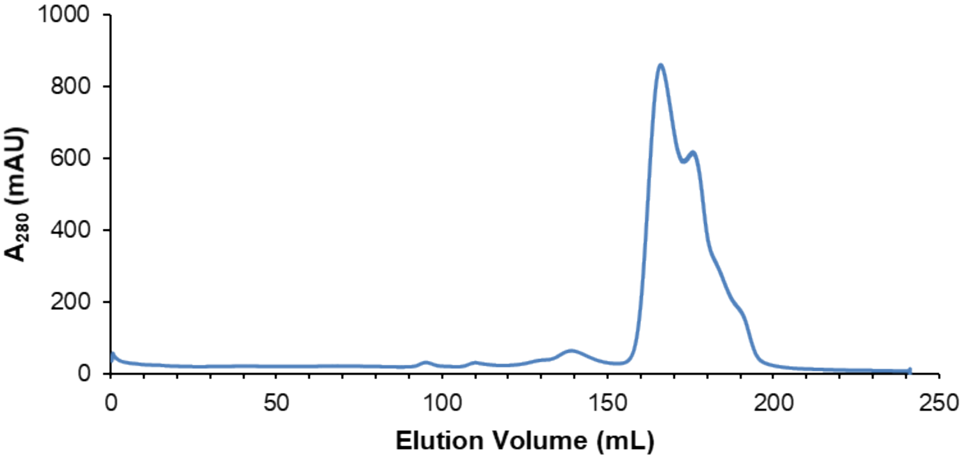

C

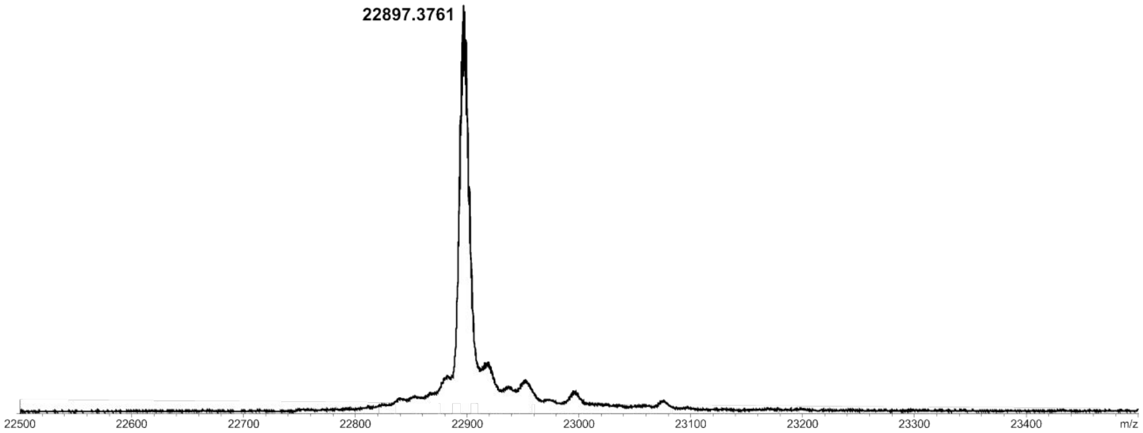

Figure S17: SDS-PAGE gel of nickel affinity purification of WTSrtA, ran alongside a protein ladder (NEB protein standard). WT = wash through, E = elution fraction. B) Chromatogram trace of elution from SEC column. Fractions containing elution volume 180-220 mL were collected and concentrated C) HRMS of fractions showed presence of WTSrtA. Expected mass 22898 Da

A

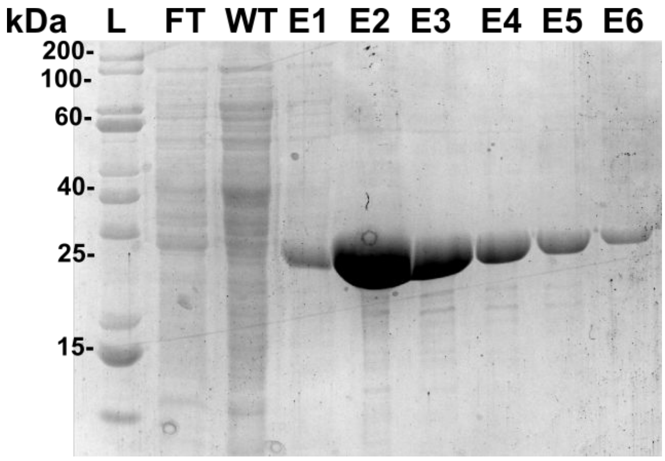

B

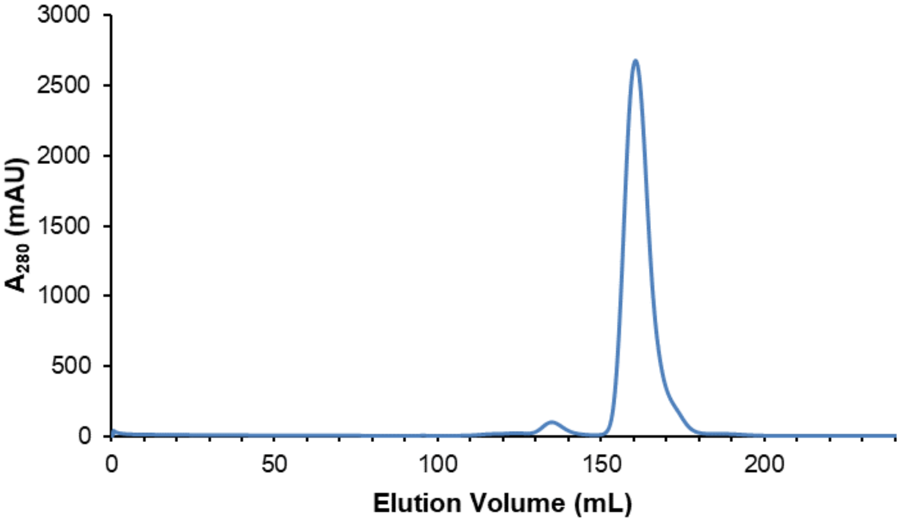

C

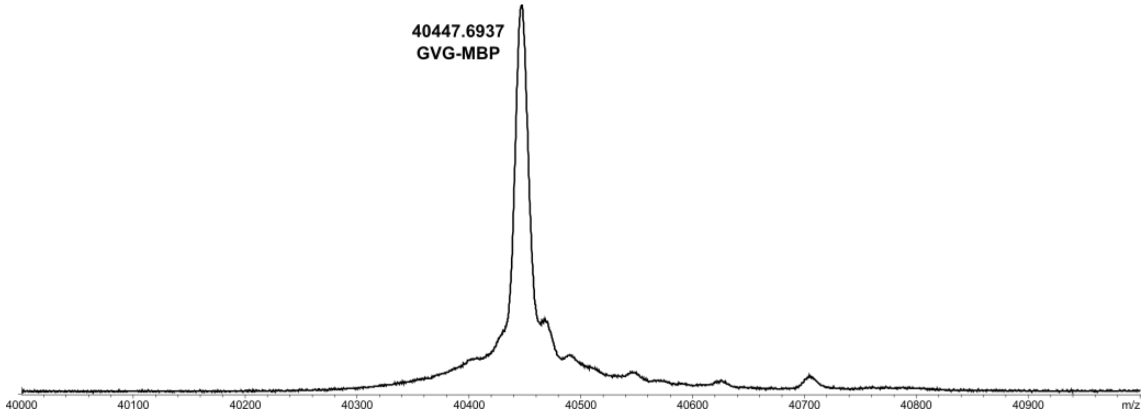

Figure S18: SDS-PAGE gel of amylose affinity purification of GVG-MBP, ran alongside a protein ladder (NEB protein standard). WT = wash through, E = elution fraction. B) Chromatogram trace of elution from SEC column. Fractions containing elution volume 150-180 mL were collected and concentrated C) HRMS of fractions showed presence of GVG-MBP. Expected mass (including action of methionine aminopeptidase) 40450 Da

A

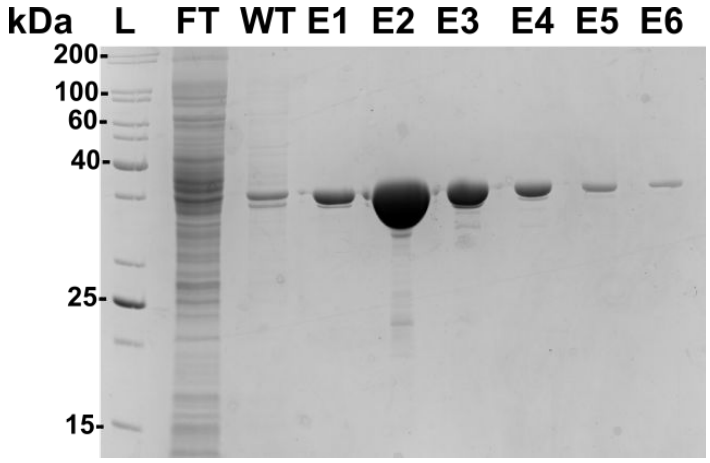

B

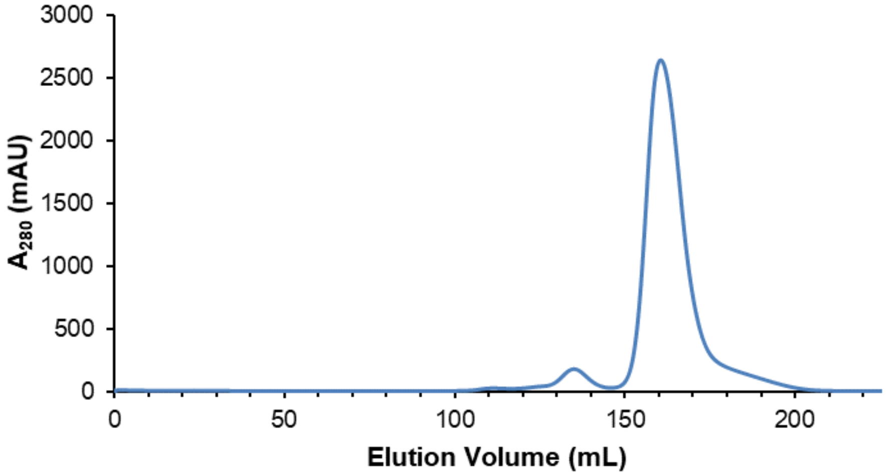

C

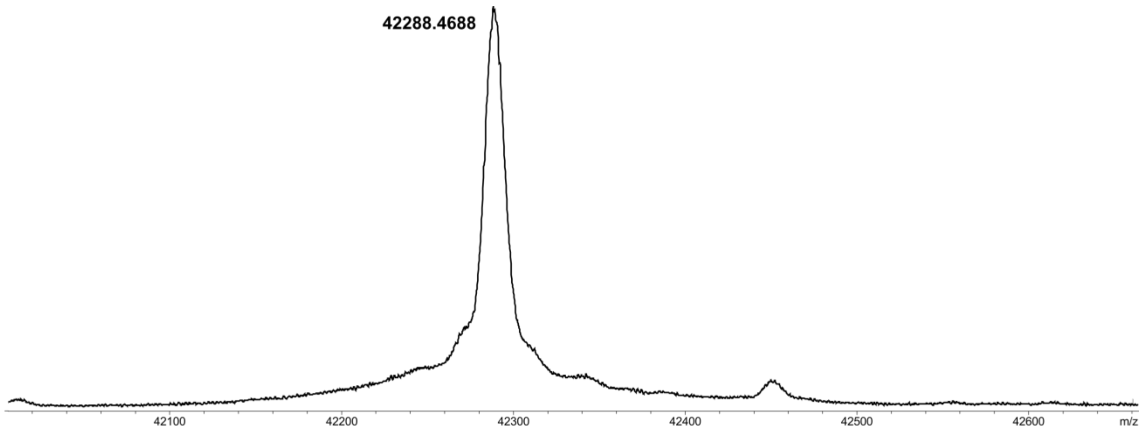

Figure S19: SDS-PAGE gel of amylose affinity purification of GVG-MBP(lins), ran alongside a protein ladder (NEB protein standard). WT = wash through, E = elution fraction. B) Chromatogram trace of elution from SEC column. Fractions containing elution volume 150-180 mL were collected and concentrated C) HRMS of fractions showed presence of GVG-MBP(ins). Expected mass 42290 Da

Synthesis of depsipeptide and peptide substrates

Synthesis of ToG and SoG building blocks ToG was synthesised in two steps described by Williamson *et al.*[5] and illustrated in Scheme S1 The synthesis involves the alkylation of Fmoc-threonine(tBu) with benzyl 2-bromoacetate, aided by coupling reagent tetrabutylammonium iodide (TBAI) to yield benzyl-protected ToG. This was followed by palladium-catalysed hydrogenolysis to deprotect the carboxyl group yielding the final product. The synthesis of SoG was carried out in the same way as it was for ToG but using an Fmoc-serine(tBu), rather than an Fmoc-threonine(tBu), as shown in Scheme S2.

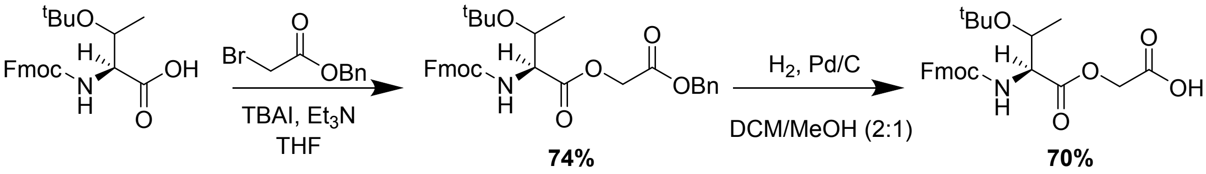

Scheme S1: Synthetic route to the ToG depsipeptide building block

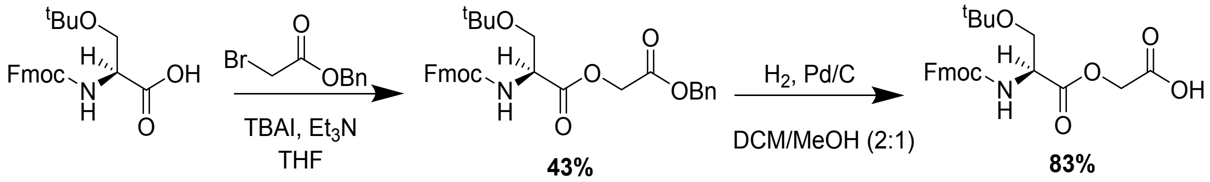

Scheme S2: Synthetic route to the SoG depsipeptide building block

Chemical synthesis of fluorescent tag A dansyl fluorophore was coupled to substrates used in sortase-labelling reactions analysed via SDS-PAGE. This enables visualisation of the labelled protein by UV illumination. In order to incorporate the fluorophore into the substrates, dansyl chloride was coupled to Fmoc-protected lysine in a Schotten–Baumann reaction, (Scheme S3).[6]

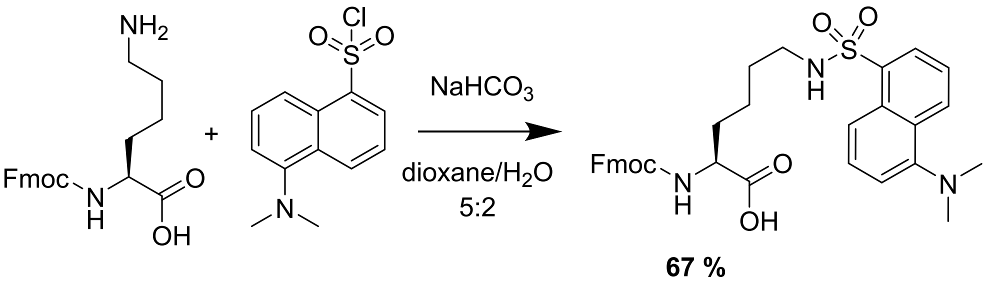

Scheme S3: Synthetic route to the Fmoc-protected dansyl lysine residue

2-(benzyloxy)-2-oxoethyl 2-((((9H-fluoren-9-yl)methoxy)carbonyl)amino)-3-(tert-butoxy)butanoate Fmoc-Thr(OtBu)-Gc-OBn.[5] **2a**

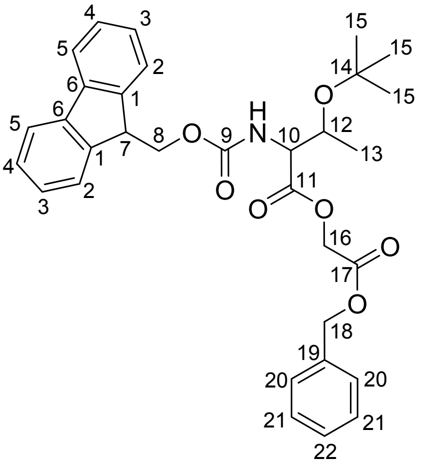

Fmoc-Thr(OtBu)-OH (10 g, 25 mmol), was dissolved in 20 mL of THF. Benzyl 2-bromoacetate (5.9 mL, 38 mmol), TBAI (3.7 g, 10 mmol) and triethylamine (5.2

mL, 30 mmol) were added sequentially and the reaction was stirred overnight at room temperature. The reaction mixture was then washed with H_2O (200 mL) and the crude product extracted with ethyl acetate (2 x 200 mL). The ethyl acetate layers were combined and washed with sodium thiosulfate solution (10% *w/v*) (2 x 400 mL) and sodium chloride solution (40% *w/v*) (400 mL). The ethyl acetate layers were combined, dried with sodium sulfate and concentrated to yield a yellow oil. Purification was carried out *via* flash column chromatography in a 4:1 (*v/v*) hexane/ethyl acetate solvent system that was increased to 1:1 hexane/ethyl acetate once product was detected. The product was concentrated *in vacuo* and dried on the high vacuum line overnight. This produced a glassy solid which yielded a colourless powder when scratched (10.2 g, 74%). **R_F**: 0.4 (2:1 (*v/v*) hexane/ethyl acetate); **^{1}H-NMR (500 MHz, CD_3OD):** δ = 7.80 (2H, d, J_{H5-H4} 7.5 Hz, H_5), 7.68 (2H, t, J_{H2-H3} 7.7 Hz, H_2), 7.39 (2H, t, $J_{H4-H5/3}$ 7.3 Hz, H_4), 7.36-7.29 (7H, m, H_3, H_{20-22}), 5.19 (2H, s, H_{18}), 4.78 (1H, d, $J_{H16-H16}$ 16.0 Hz, H_{16}) 4.69 (1H, d, $J_{H16-H16}$ 16.0 Hz, H_{16}), 4.42-4.35 (2H, m, H_8), 4.33 (1H, d, $J_{H10-H12}$ 3.0 Hz, H_{10}), 4.27-4.23 (2H, m, H_{12}, H_7) 1.19 (3H, d, $J_{H13-H12}$ 6.2 Hz, H_{13}), 1.15 (9H, s, H_{15}); **^{13}C-NMR (100 MHz, CD_3OD):** δ = 170.4 (CO), 167.5 (CO), 157.3 (CO), 143.8 (C_1/C_6), 141.2 (C_1/C_6), 135.4 (C_{19}), 128.2 (ArC), 128.0 (ArC), 127.9 (ArC), 127.4 (ArC), 126.8 (ArC), 124.9 (C_2), 119.6 (C_5), 74.0 (C_{14}), 67.5 (C_{12}) 66.8 ($C_{18/8}$), 66.6 ($C_{18/8}$) 61.0 (C_{16}), 59.8 (C_{10}), 47.0 (C_7) 27.5 (C_{15}), 19.5 (C_{13}); **IR (V_{max}/cm^{-1}):** 3436.39 (NH); 2975.98 (CH); 1772.95, 1757.40, 1724. 84 (C=O).

2-((N-(((9H-fluoren-9-yl)methoxy)carbonyl)-O-(tert-butyl)threonyl)oxy)acetic acid Fmoc-Thr(OtBu)-Gc-OH.[5] **3a**

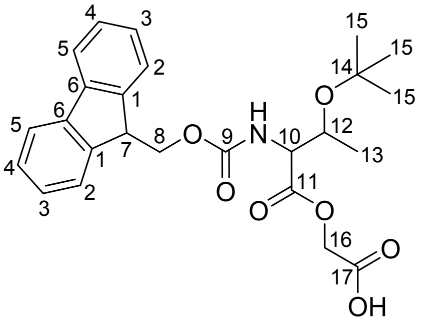

Fmoc-Thr(OtBu)-Gc-OBn (1.6 g, 2.93 mmol) was dissolved in 8 mL methanol/ 4 mL DCM before H_2O (4 mL) was slowly added. To the stirred solution, Pd/C (10%) (160 mg) was added and the reaction mixture was stirred under a H_2 atmosphere for 1 hour 45 minutes. The reaction mixture was filtered through celite and washed with methanol (~200 mL). The crude product was concentrated, freeze dried to remove excess water and purified via flash column chromatography (9:1 (*v/v*) DCM/ EtOAc, 1% acetic acid). The product was concentrated to yield a foamy colourless solid (938 mg, 70%). **R_F**: 0.59 (9:1 (*v/v*) DCM/ EtOAc, 1% acetic acid); **^{1}H-NMR (400 MHz, CD_3OD):** δ = 7.80 (2H, d, J_{H5-H4} 7.5 Hz, H_5), 7.68 (2H, t, J_{H2-H3} 7.5 Hz, H_2), 7.39 (2H, t, $J_{H4-H5/3}$ 7.5 Hz, H_4), 7.31 (2H, t, $J_{H3-H4/2}$, H_3), 4.72 (1H, d, $J_{H16-H16}$ 16.0 Hz, H_{16}) 4.58 (1H, d, $J_{H16-H16}$ 16.0 Hz, H_{16}), 4.39 (2H, dd, J_{H8-H7} 6.9 Hz, J_{H8-H2} 2.4 Hz, H_8), 4.33 (1H, d, $J_{H10-H12}$ 3.0 Hz, H_{10}), 4.31-4.24 (2H, m, H_{12}, H_7) 1.20 (3H, d, $J_{H13-H12}$ 6.2 Hz, H_{13}), 1.16 (9H, s, H_{15}); **^{13}C-NMR (100 MHz, CD_3OD):** δ = 170.3 (CO), 169.3 (CO), 157.3 (CO), 143.8 (C_1/C_6), 141.2 (C_1/C_6), 127.4 (C_4), 126.8 (C_3), 124.9 (C_2), 119.5 (C_5), 74.0 (C_{14}), 67.5 (C_{12}) 66.8 (C_8), 60.7 (C_{16}), 59.8 (C_{10}), 47.0 (C_7) 27.4 (C_{15}), 19.5 (C_{13}); **IR (V_{max}/cm^{-1}):** 3449.26 (OH); 3357.70 (NH); 2977.08 (CH); 1751.75, 1707.91 (C=O); **HRMS (ES):** $C_{25}H_{29}NO_7$ $[M+Na]^+$ 478.1836, found $[M+Na]^+$ 478.1844.

N2-(((9H-fluoren-9-yl)methoxy)carbonyl)-N6-((5-(dimethylamino)naphthalen-1-yl)sulfonyl)lysine Fmoc-K(dansyl)-COOH[7]

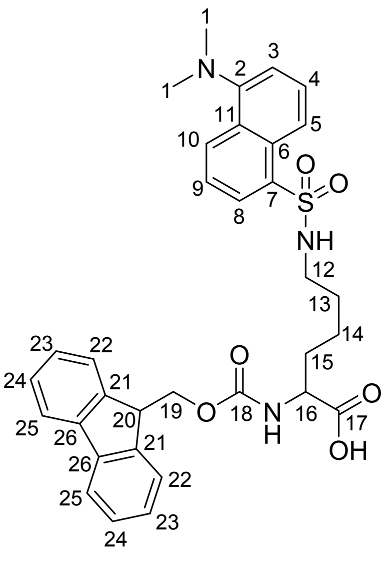

Sodium hydrogen carbonate (978 mg, 11.64 mmol) was added to a solution of Fmoc-Lys-OH (1.5g, 4.07 mmol) and dansyl chloride (1.65g, 6.12 mmol) in 5:2 dioxane/H_2O (140 mL) and stirred overnight at room temperature. The reaction mixture was then diluted with ethyl acetate (100 mL) and washed with 5% aq. $KHSO_4$ (200 mL). The ethyl acetate was collected and aqueous later further extracted with ethyl acetate (100 mL). The organic layers were combined, dried with magnesium sulfate and concentrated. The product was purified via flash column chromatography (9:1 (*v/v*) DCM/MeOH, 1% AcOH). The product was concentrated and dried on the high vacuum line overnight to yield a fluorescent yellow foamy solid (1.65g, 67%). **R_F**: 0.53 (9:1 (*v/v*) DCM/MeOH, 1% acetic acid); **IR (V_{max}/cm^{-1}):** 3270.12 (OH); 2940.04, 2866.81, 2789.66 (CH); 1703.79 (C=O); **HRMS (ES):** $C_{33}H_{35}N_3O_6S$ $[M]^+$ 602.2319, found $[M]^+$ 602.2319

Solid phase peptide synthesis

General procedure for SPPS of depsipeptides

Coupling depsipeptide building block onto solid phase resin

Step 1: The H-Gly-2-ClTrt resin (1.1 mmol/g loading) was swollen in DMF for 20 minutes.

Step 2: Oxymapure (5 eq.) and the depsipeptide building block (5 eq.) were dissolved in DMF (6 mL), followed by the addition of DIC (5 eq.). The solution was transferred to the swollen resin and the mixture was left to spin for 1 hr at room temperature. The resin was isolated by filtration and washed with DMF (3 x 6 mL x 2 min spins).

Step 3: Step 2 was repeated for double coupling of the depsipeptide building block

Step 4: A capping solution (50 μL Ac_2O, 203 μL DIPEA in 6 mL DMF) was added to the resin and the mixture was left to spin for 1 hr at room temperature. The resin was isolated by filtration and washed with DMF (3 x 6 ml x 2 min spins).

Fmoc deprotection and peptide elongation

Step 5: The Fmoc-building block-resin was treated with piperidine (20% in DMF) (2 x 6 mL x 5 min spins) and then washed with DMF (3 x 6 mL x 2 min spins).

Step 6: The peptide sequence was elongated by repeating step 2, with an Fmoc-amino acid (5 eq.) instead of the depsipeptide building block, followed by step 5. This was repeated until the desired peptide sequence was achieved.

Cleavage and Isolation

Step 7: The amino acid-resin was washed with DMF (3 x 6 mL x 2 min spins), DCM (3 x 6 mL x 2 min spins) and methanol (3 x 6 mL x 2 min spins).

Step 8: The resin was isolated by filtration before being dried under nitrogen. A cleavage cocktail (2.85 mL TFA, 75 μL H_2O, 75 μL IPS) was added to the resin and left to spin for 2 hr at room temperature to simultaneously cleave the peptide from the resin and deprotect side chains.

Step 9: The resin was filtered into cold diethyl ether (40 mL) to precipitate the protein. The precipitate was pelleted by centrifugation (4000 x g, 10 min) and the diethyl ether discarded. The peptide pellet was resuspended in cold diethyl ether (40 mL) and the process repeated three times. The pellet was then dried under nitrogen.

Step 10: The peptide was dissolved in minimal amount of water and freeze dried and analysed by LCMS.

General procedure for synthesis of peptides

Peptides that did not require incorporation of the depsipeptide building block were synthesised using a CEM Liberty Blue™ Peptide Synthesiser with microwave assistance. The synthesis was carried out on a 0.25 mmol scale using a H-Gly-2-ClTrt resin (1.1 mmol/g loading capacity), Oxyma and DIC in DMF as coupling agents and 20% piperidine in DMF for deprotection. Amino acids were used at a concentration of 0.2 M. Double couplings were used for each amino acid at 90 ^{o}C for 2 minutes using manufacturer preset coupling cycles. Unnatural amino acids (dansyl-lysine) were set to couple at a reduced temperature of 50 ^{o}C for 10 minutes. After the final amino acid coupling and deprotection, cleavage and isolation were carried out manually following the procedure as per the depsipeptide synthesis.

Analytical Data

*^{1}H and ^{13}C NMR spectra for **2b** (CD_3OD)*

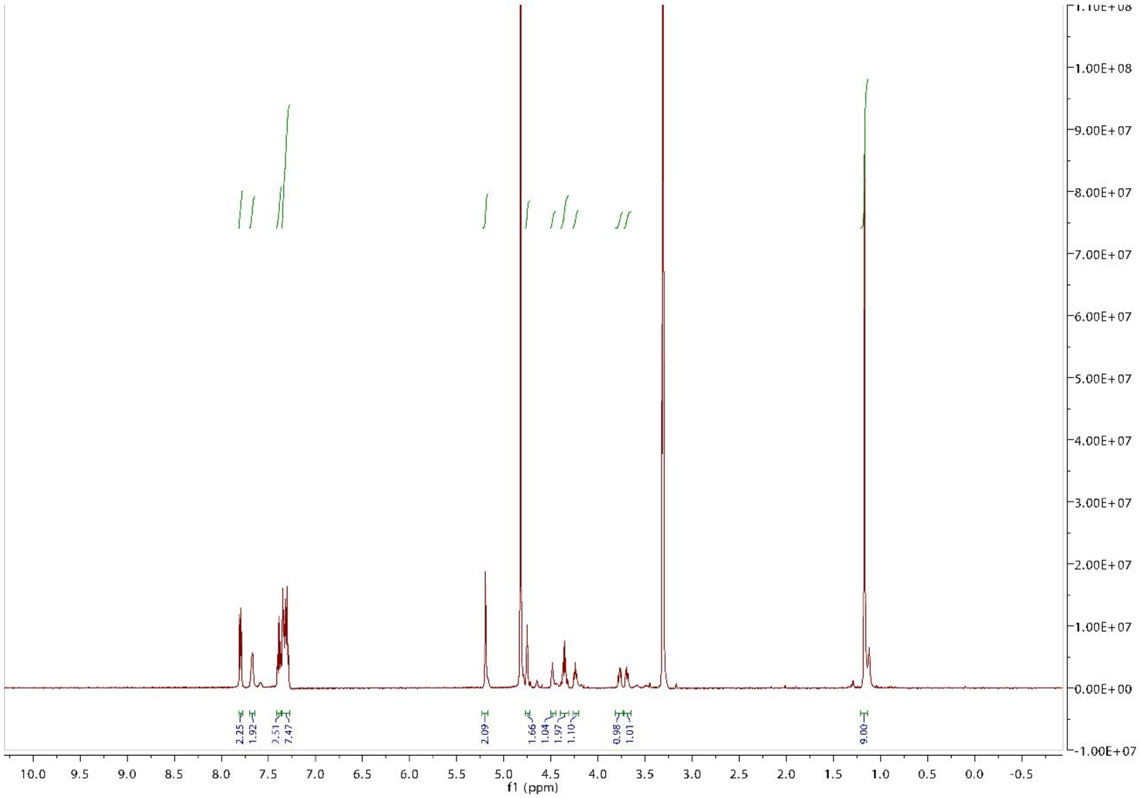

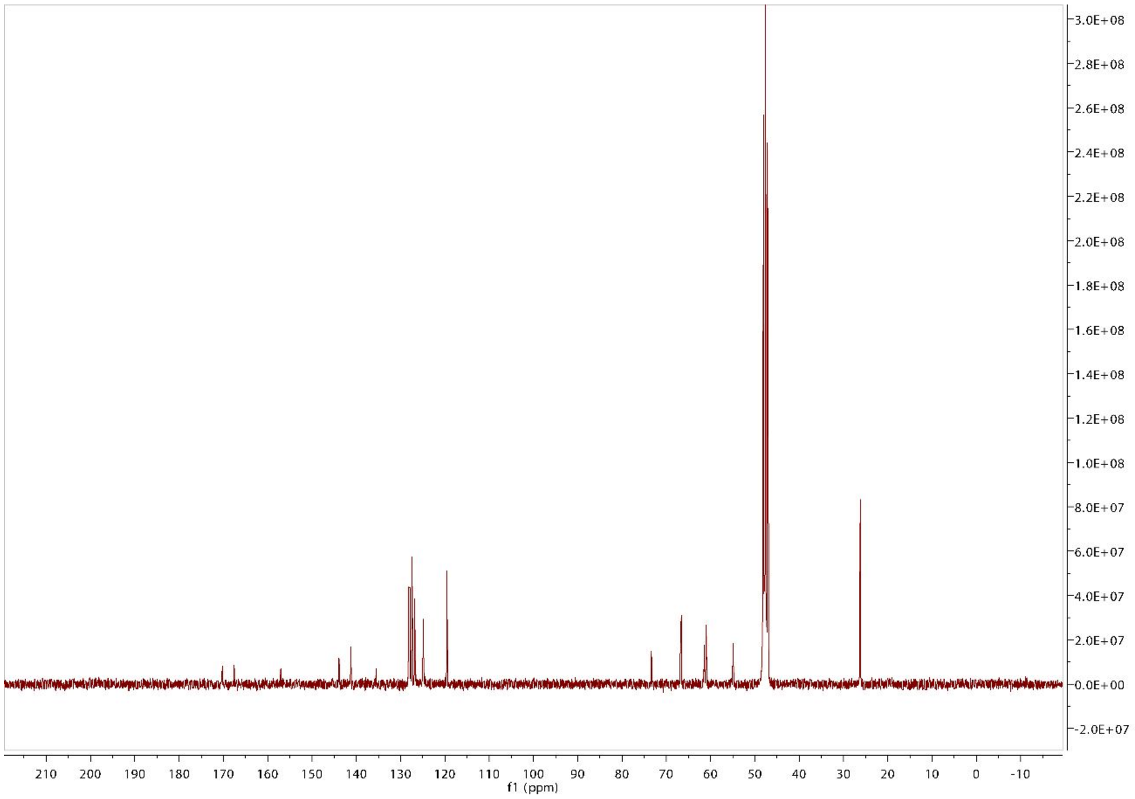

^1H and ^{13}C NMR spectra for **3b** (CD_3OD)

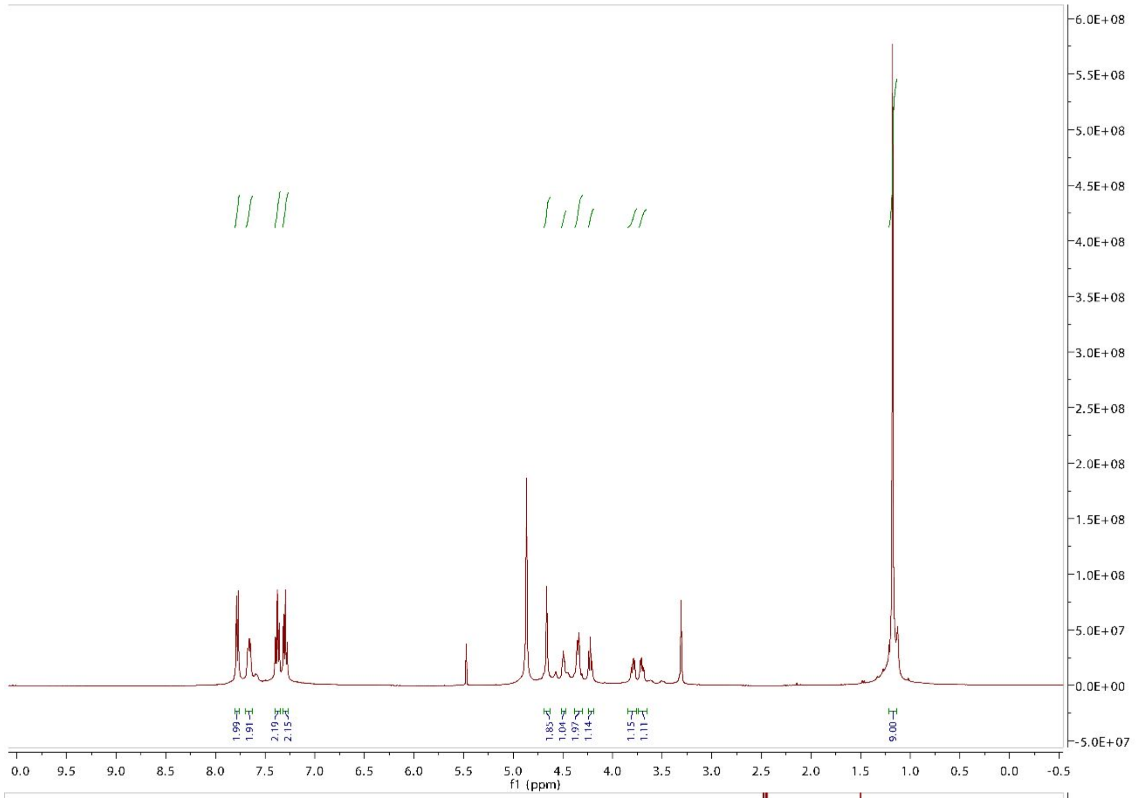
1.99
1.91
2.19
2.15
1.85
1.04
1.97
1.14
1.15
1.11
9.00
f1 (ppm)

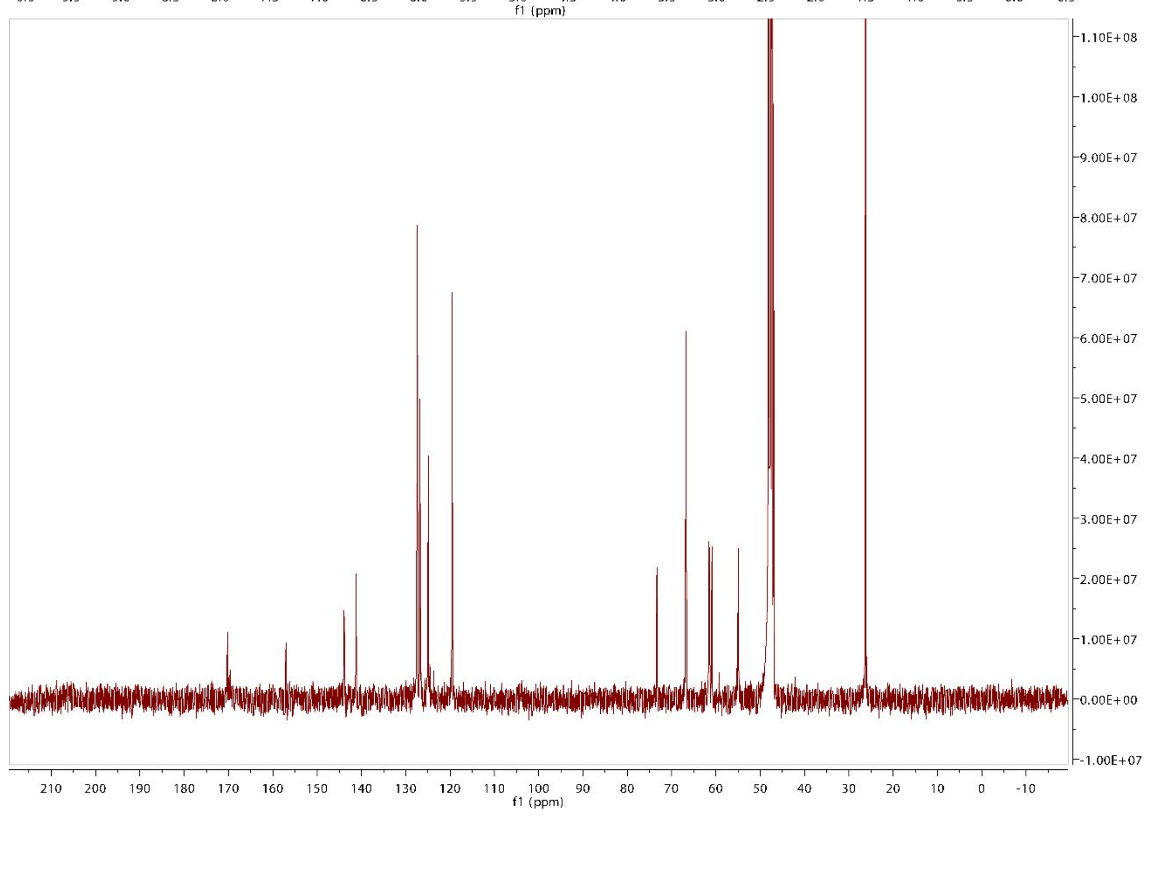
f1 (ppm)

*^{1}H spectrum for **3b** ($CDCl_3$)*

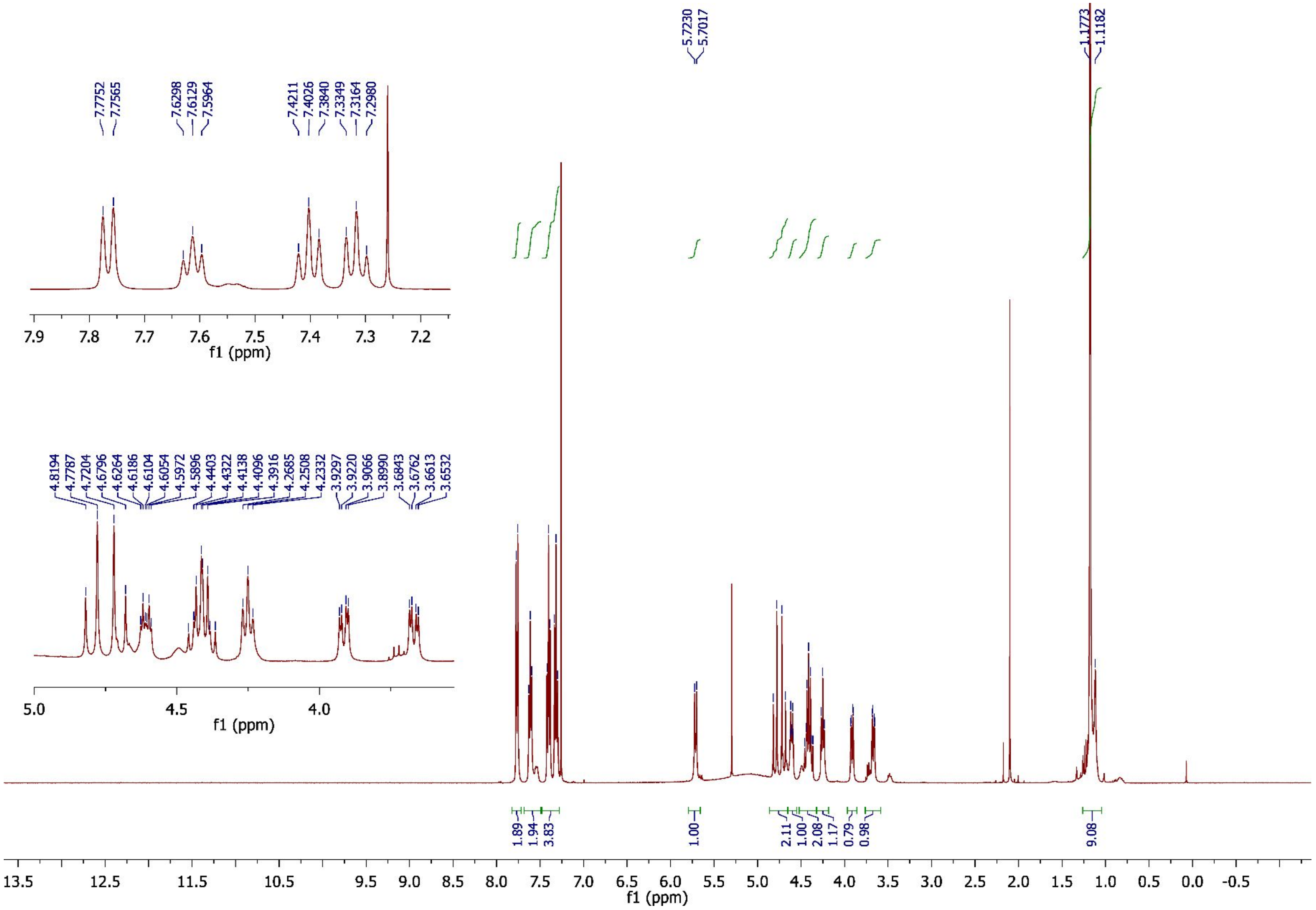

7.7752
7.7565
7.6298
7.6129
7.5964
7.4211
7.4026
7.3840
7.3349
7.3164
7.2980
7.9 7.8 7.7 7.6 7.5 7.4 7.3 7.2
f1 (ppm)
4.8194
4.7787
4.7204
4.6796
4.6264
4.6186
4.6104
4.6054
4.5972
4.5896
4.4403
4.4322
4.4138
4.4096
4.3916
4.2685
4.2508
4.2332
3.9297
3.9220
3.9066
3.8990
3.6843
3.6762
3.6613
3.6532
5.0 4.5 4.0
f1 (ppm)
5.7230
5.7017
1.1773
1.1182
1.89
1.94
3.83
1.00
2.11
1.00
2.08
1.17
0.79
0.98
9.08
13.5 12.5 11.5 10.5 9.5 9.0 8.5 8.0 7.5 7.0 6.5 6.0 5.5 5.0 4.5 4.0 3.5 3.0 2.5 2.0 1.5 1.0 0.5 0.0 -0.5
f1 (ppm)

H_2N-K(Dansyl)ALPEToGG-COOH

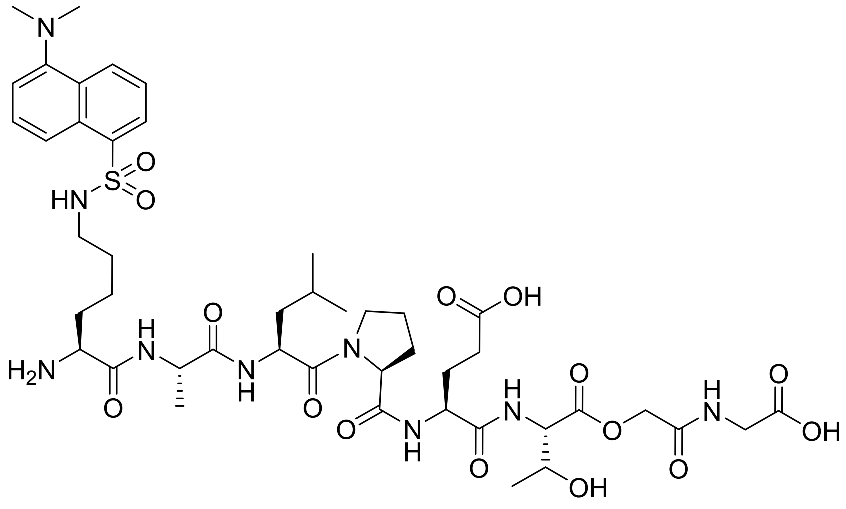

Yield: 31.3 mg, 18.9 %**; HRMS (ES):** $C_{45}H_{67}N_9O_{15}S$ [M^{2+}] 504.2358, found [M^{2+}] 504.2345 **LC** detection wavelength 280 nm

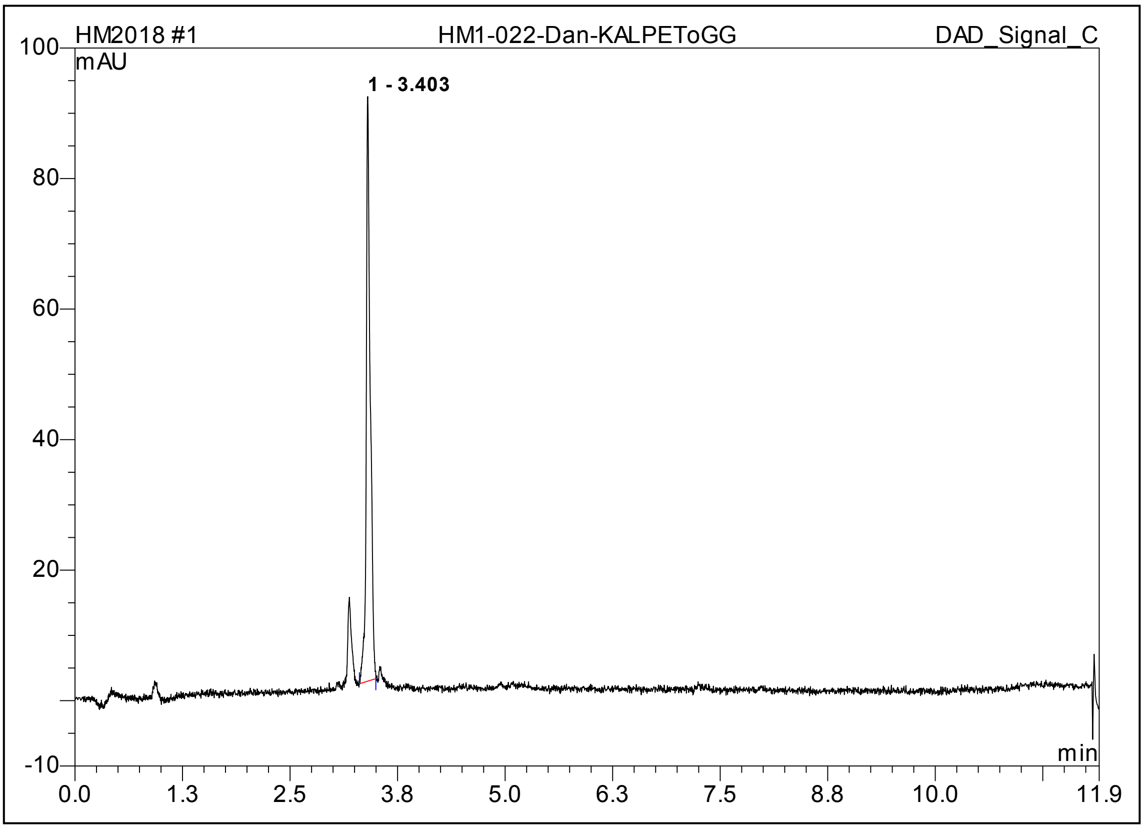

H_2N-K(Dansyl)ALPESoGG-COOH

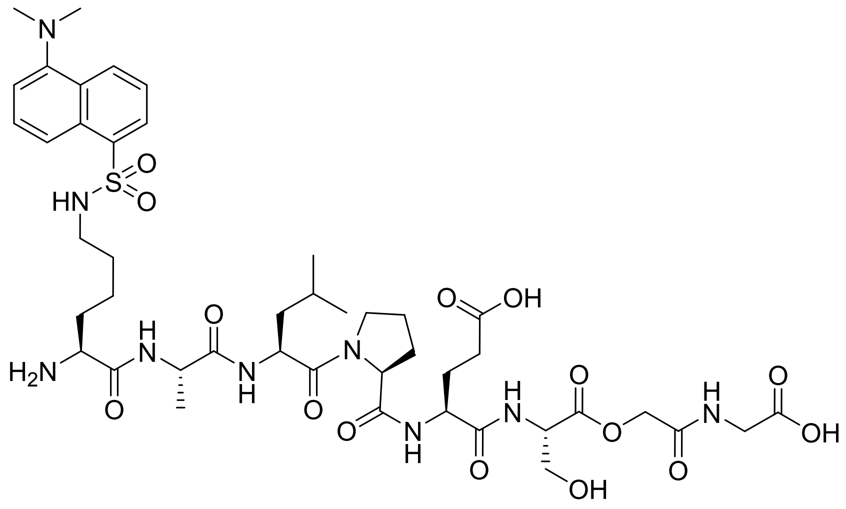

Yield: 48.6 mg, 29.7 %**; HRMS (ES):** $C_{44}H_{65}N_9O_{15}S$ [M^{2+}] 496.7144, found [M^{2+}] 496.7153 **LC** detection wavelength 280 nm

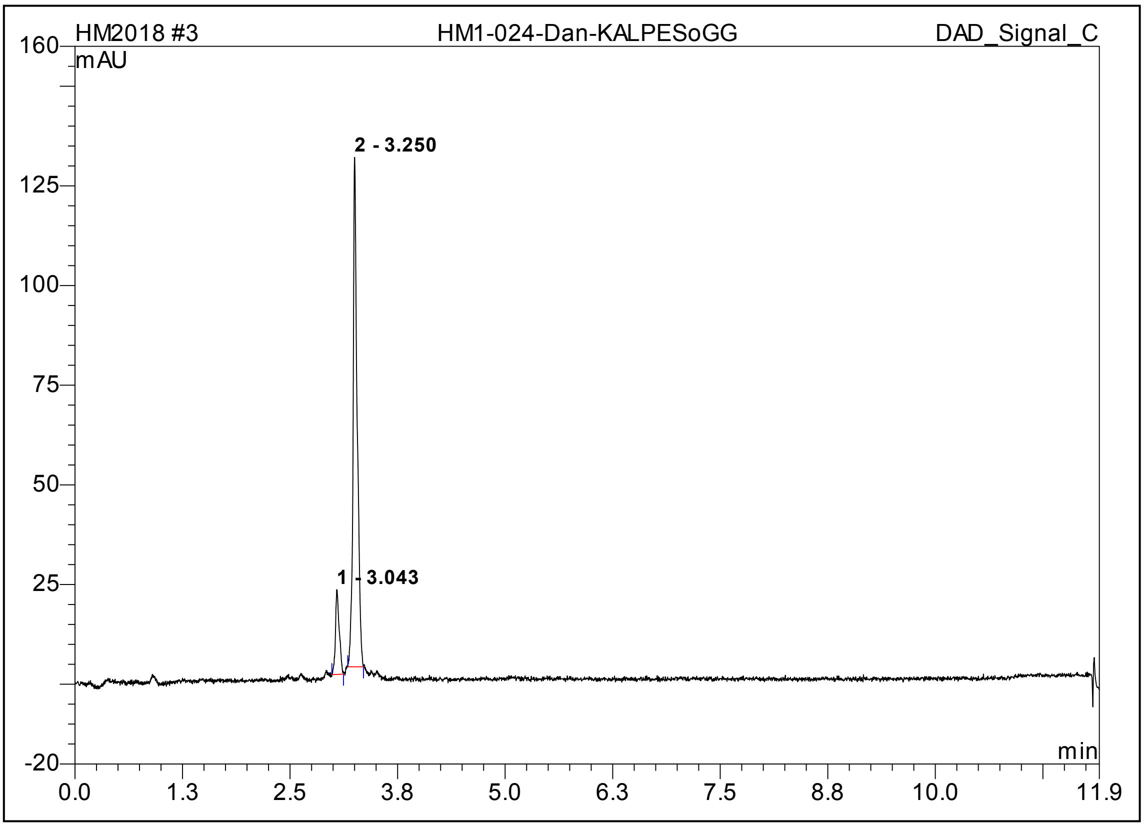

H_2N-K(Dansyl)ALAEToGG-COOH

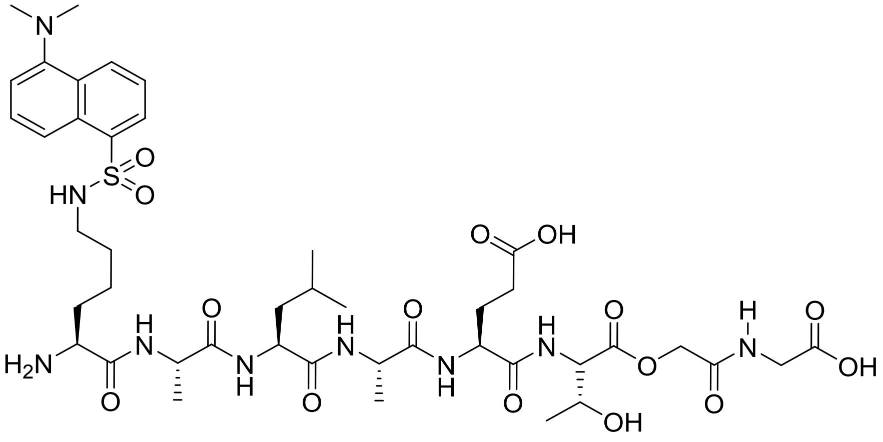

Yield: 120.6 mg, 74.6 %**; HRMS (ES):** $C_{43}H_{65}N_9O_{15}S$ [M^{2+}] 490.7160, found [M^{2+}]: 490.7154 **LC** detection wavelength 280 nm

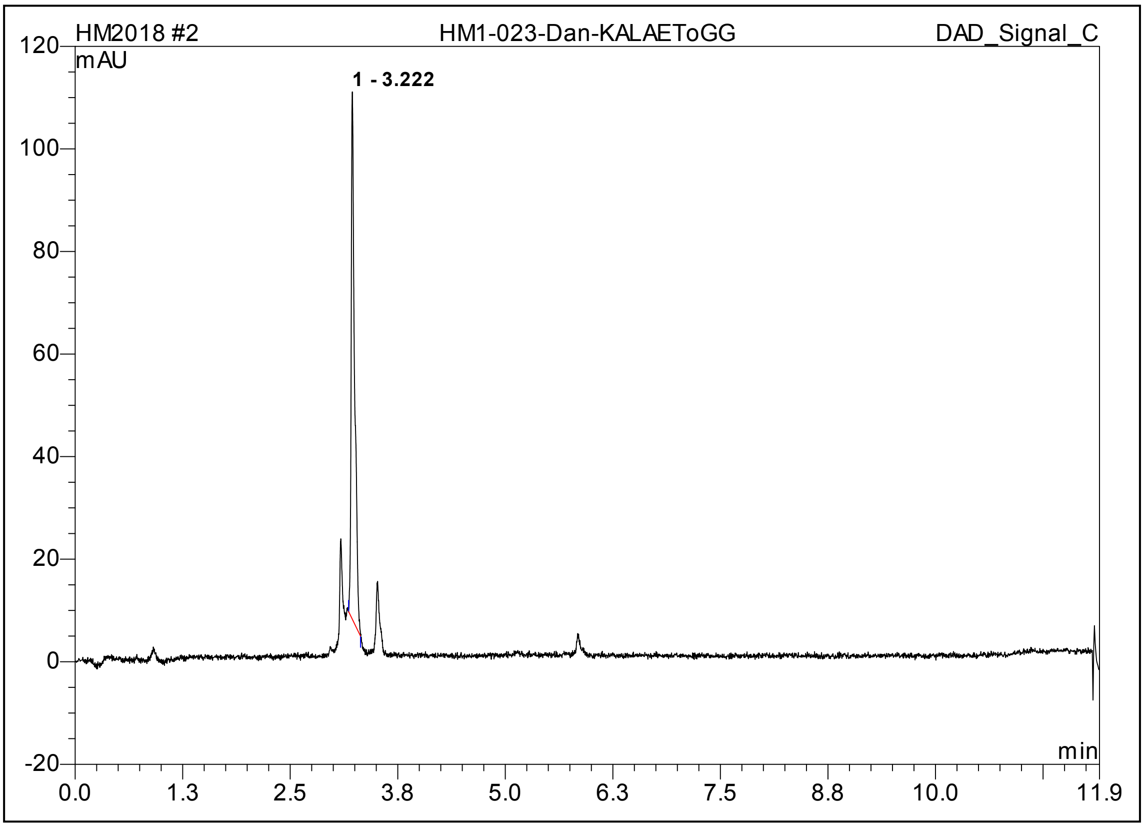

H_2N-K(Dansyl)ALPETGG-COOH

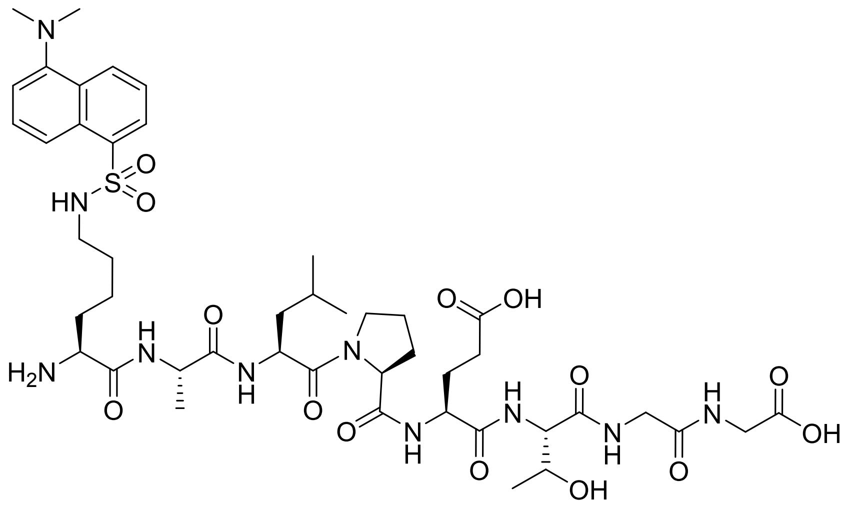

Yield: 58.1 mg, 57.8 %; **HRMS (ES):** $C_{45}H_{68}N_{10}O_{14}S$ [M^+] 1005.1550, found [M^{2+}] 503.2421 **LC** detection wavelength 280 nm

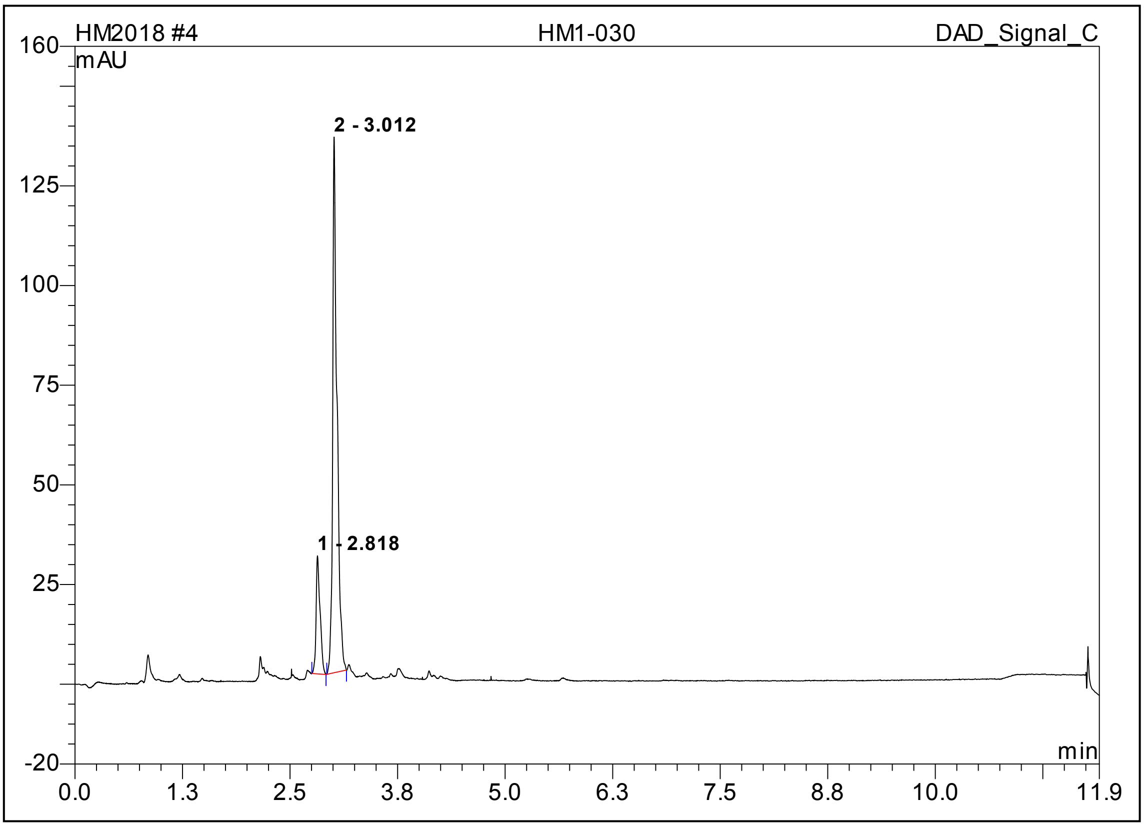

H_2N-K(Dansyl)ALPESGG-COOH

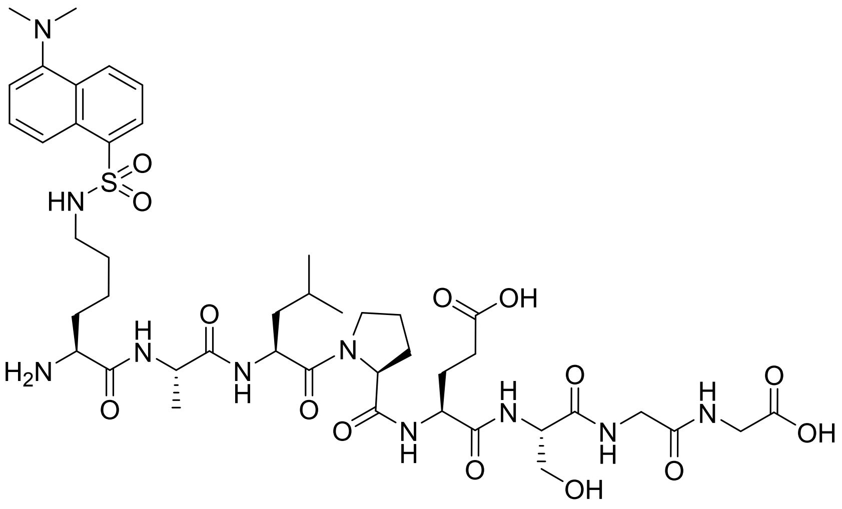

Yield: 58.7 mg, 59.2 %; **HRMS (ES):** $C_{44}H_{66}N_{10}O_{14}S$ [M^+] 991.1280, found [M^{2+}] 496.2335 **LC** detection wavelength 280 nm

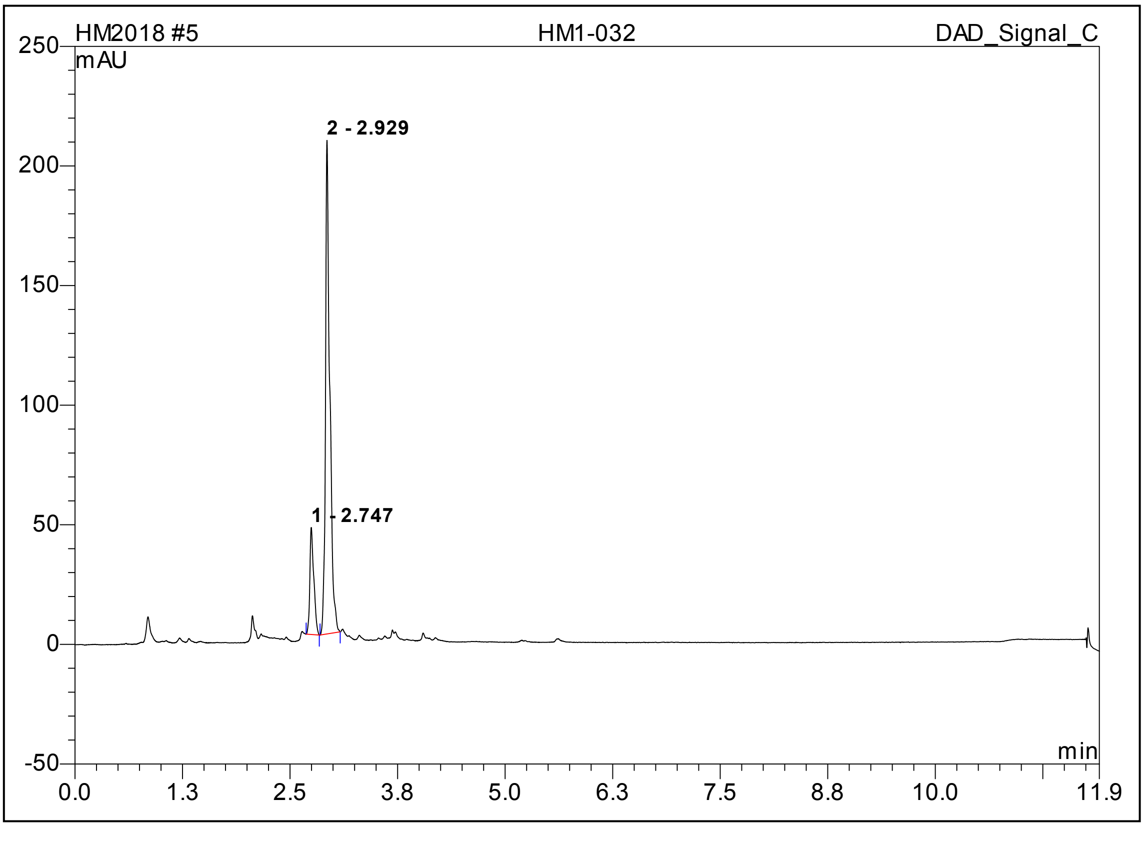

H_2N-K(Dansyl)ALAETGG-COOH

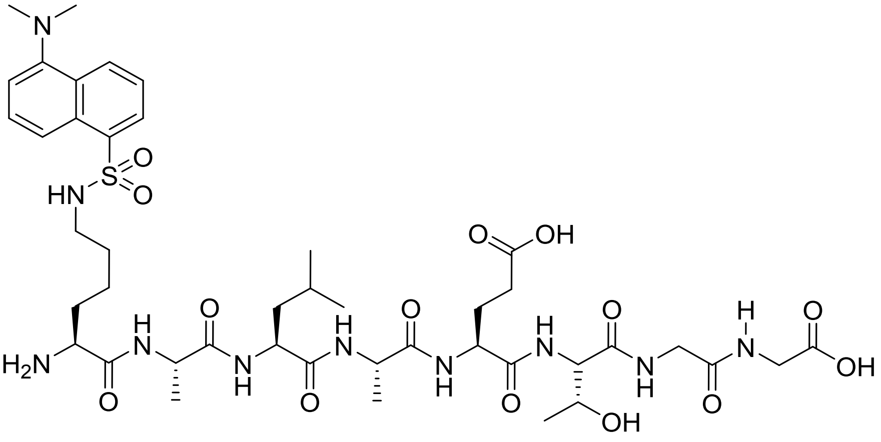

Yield: 60.6 mg, 61.1 %; **HRMS (ES):** $C_{43}H_{66}N_{10}O_{14}S$ [M^+] 979.1170, found [M^{2+}] 490.2331 **LC** detection wavelength 280 nm

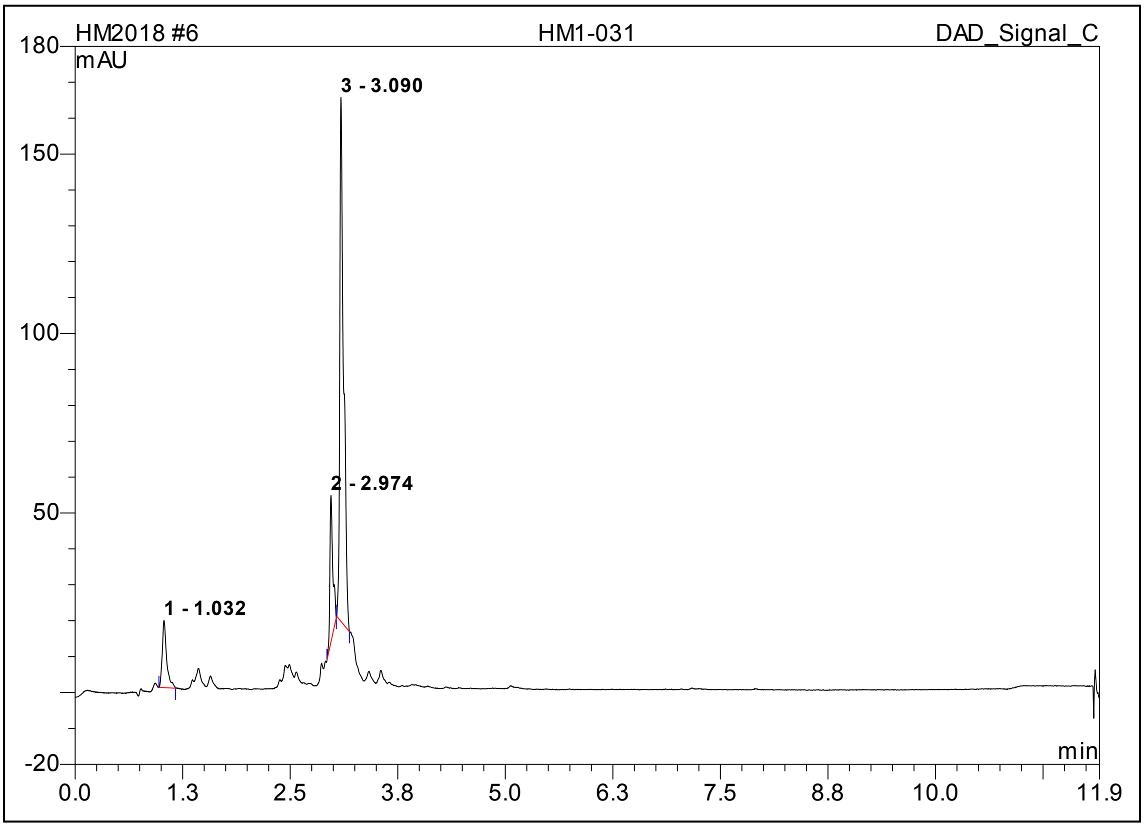

H_2N-AYLPEToGG-COOH

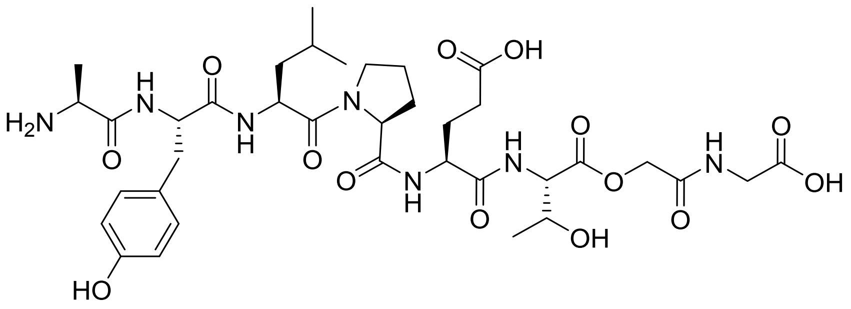

HRMS (ES): $C_{36}H_{53}N_7O_{14}$ [M^+] 808.3931, found [M^{2+}] 404.6898 and [M+Na] 830.3543 **LC** detection wavelength 254 nm

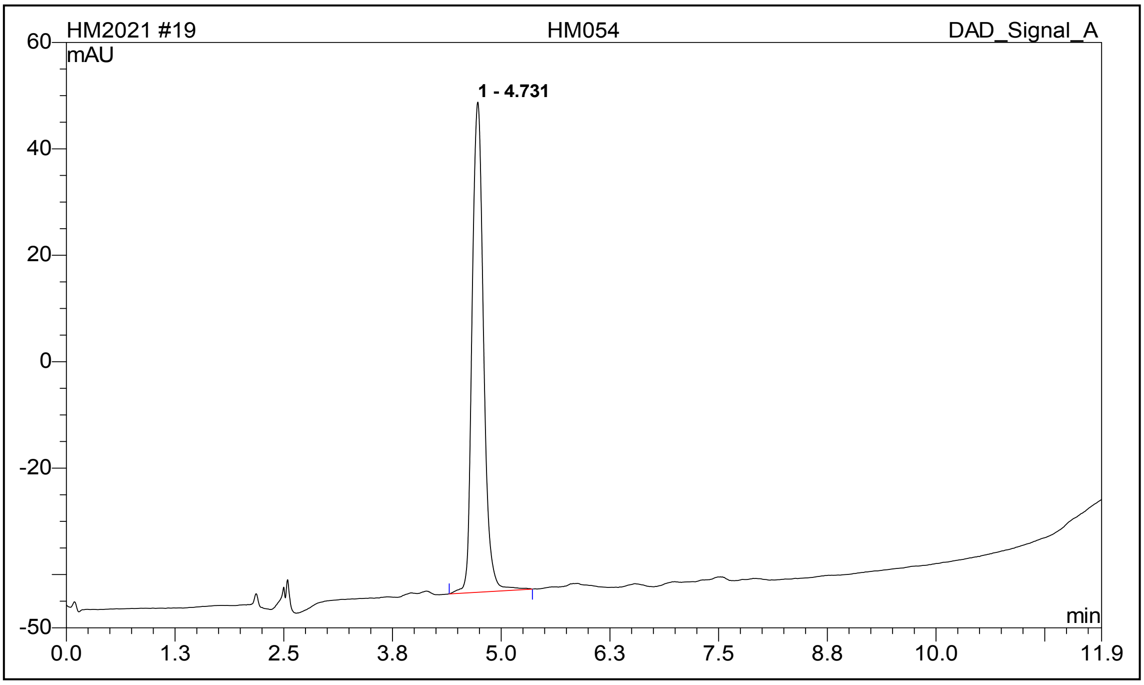

H_2N-AYLPESoGG-COOH

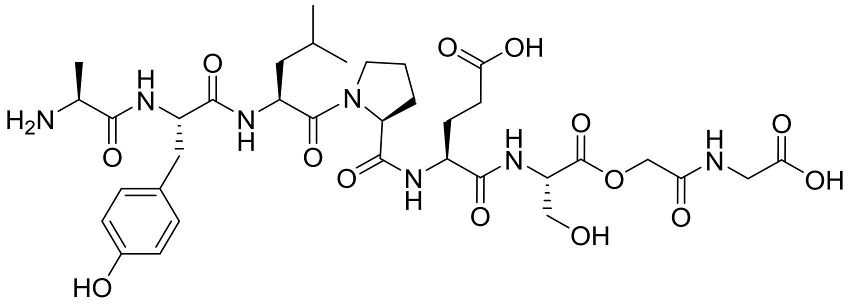

HRMS (ES): $C_{35}H_{51}N_7O_{14}$ [M^+] 794.3645, found [M^{2+}] 397.6819 **LC** detection wavelength 254 nm

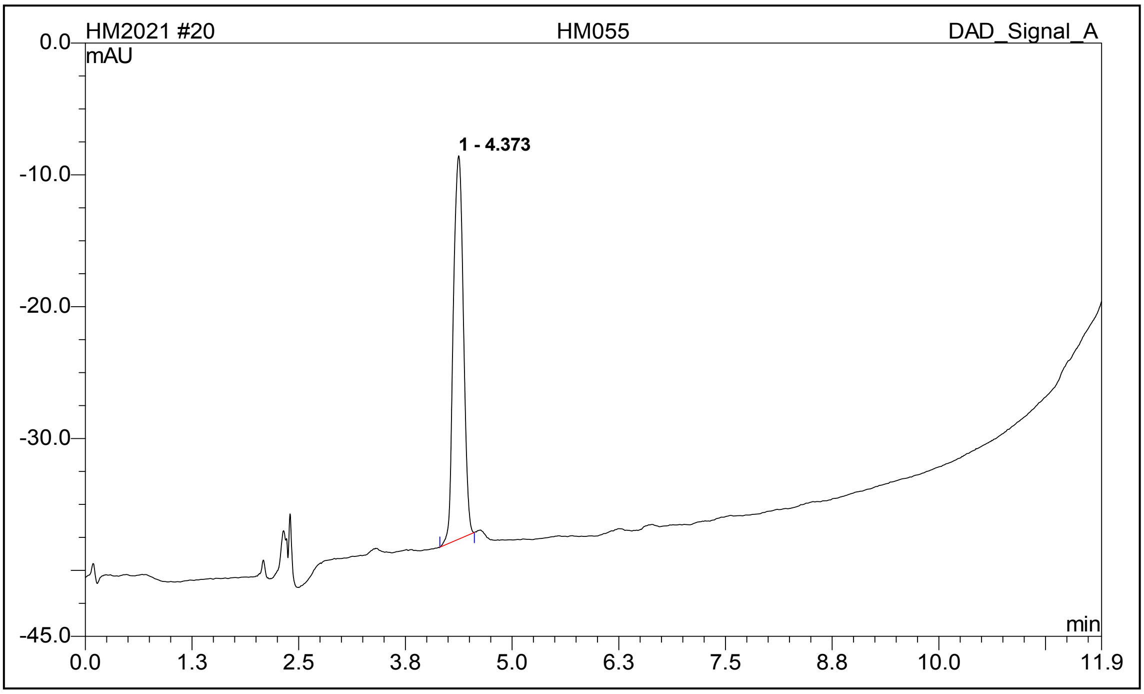

Fluorescein-GABA-YLPEToGG-COOH

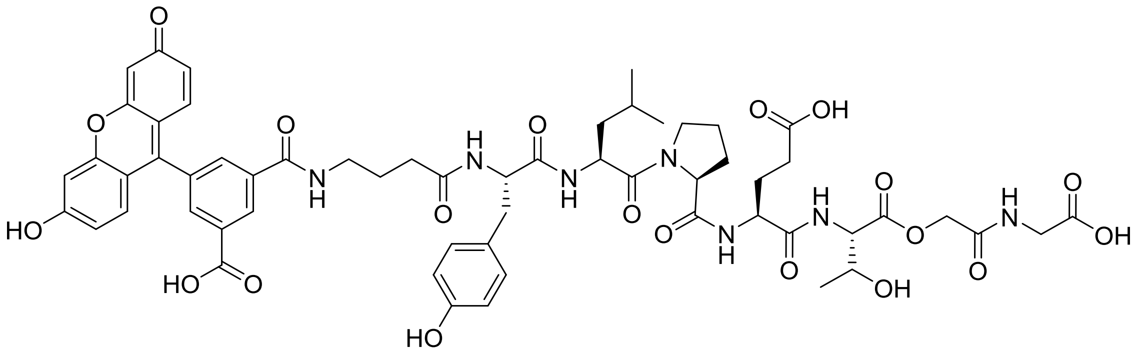

GABA-YLPEToGG was synthesised following the general procedure for SPPS of peptides. GABA-YLPEToGG (5 mg, 6.1 μmol), was dissolved in DMF (2 mL) and 5(6)-carboxy-fluorescein N-succinimidyl ester (4.3 mg, 9.1 μmol) and DIPEA (1.6 μL, 9.1 μmol) were added. The reaction was stirred at RT for 16 hr. H-Gly-2-ClTrt resin (1.1 mmol/g loading capacity, 50 mg) was added to the reaction mixture and rotated for 1 hr to remove any unreacted ester. The solution was obtained, concentrated, dissolved in dioxane/H_2O and freeze dried to yield a yellow, fluffy solid.

HRMS (ES): $C_{58}H_{65}N_7O_{20}$ [M^+] 1180.4355, found [M^+] 1180.4357 **LC** detection wavelength 254 nm

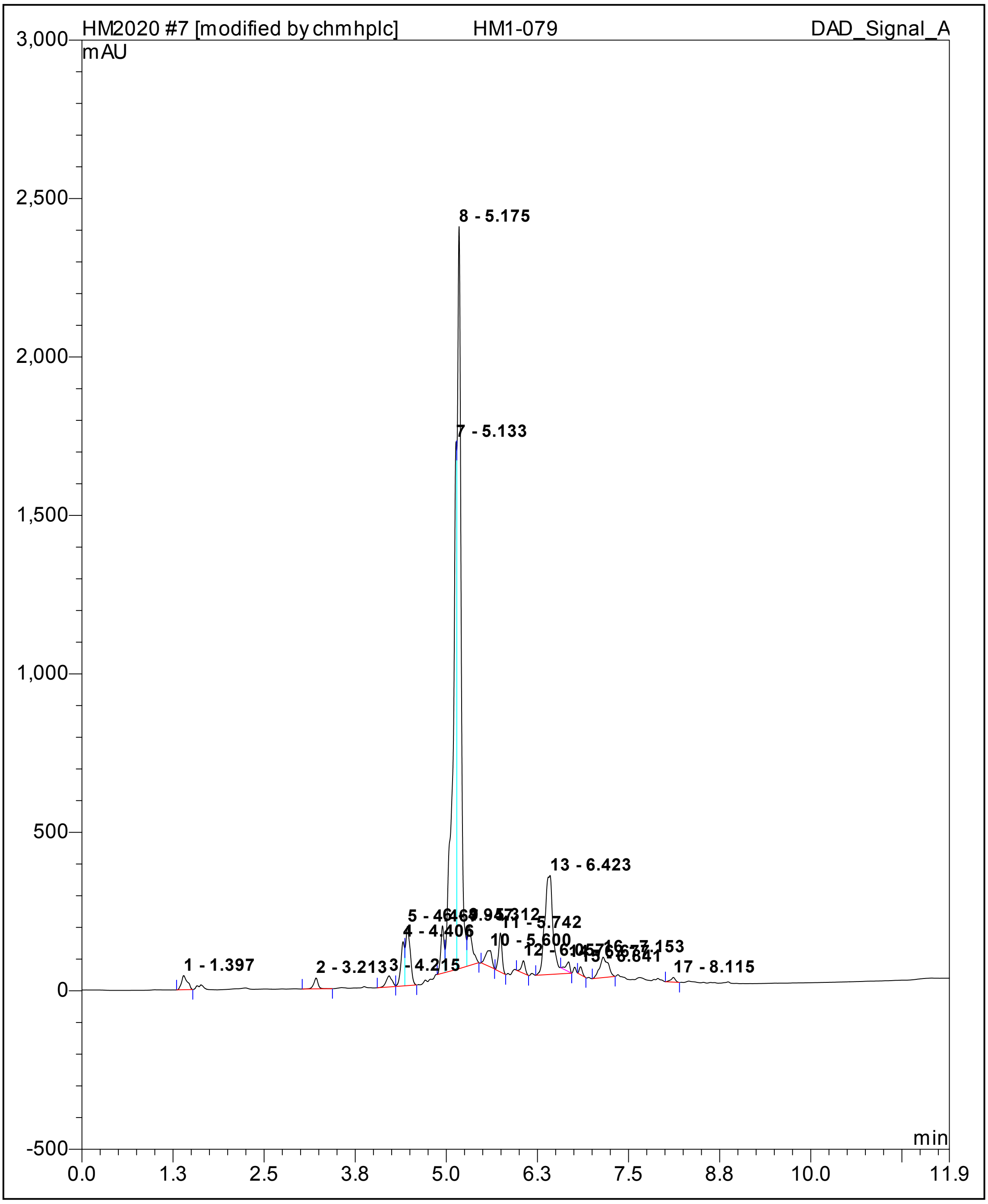
HM2020 #7 [modified by chmhplc]
HM1-079
DAD_Signal_A
mAU
3,000
2,500
2,000
1,500
1,000
500
0
-500
min
0.0
1.3
2.5
3.8
5.0
6.3
7.5
8.8
10.0
11.9
8 - 5.175
7 - 5.133
13 - 6.423
11 - 5.742
10 - 5.600
1 - 1.397
2 - 3.213
3 - 4.215
4 - 4.406
16 - 7.153
15 - 6.641
17 - 8.115

TAMRA-GABA-AVLEAYLPESoGG-COOH

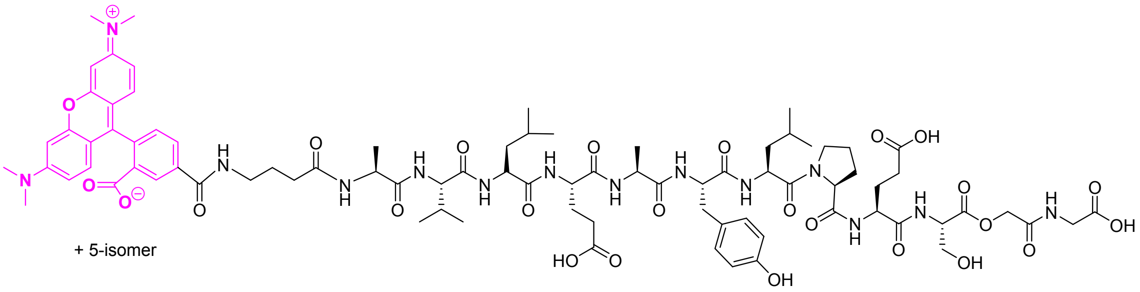

GABA-AVLEAYLPESoGG was synthesised following the general procedure for SPPS of peptides. GABA-AVLEAYLPESoGG (10 mg, 7.8 μmol), was dissolved in DMF (2 mL) and 5(6)-carboxy-tetramethylrhodamine N-succinimidyl ester (8 mg, 15.2 μmol) and DIPEA (40 μL, 0.3 mmol) were added. The reaction was stirred at RT for 16 hr. H-Gly-2-ClTrt resin (1.1 mmol/g loading capacity, 50 mg) was added to the reaction mixture and rotated for 1 hr to remove any unreacted ester. The solution was obtained, concentrated, dissolved in dioxane/H_2O and freeze dried to yield a pink, fluffy solid as a mixture of peptides containing the two isomers and trace unmodified peptide.

HRMS (ES): $C_{83}H_{114}N_{14}O_{25}$ [M^+] 1704.7976, found [M^{2+}] 852.4065 **LC** detection wavelength 254 nm

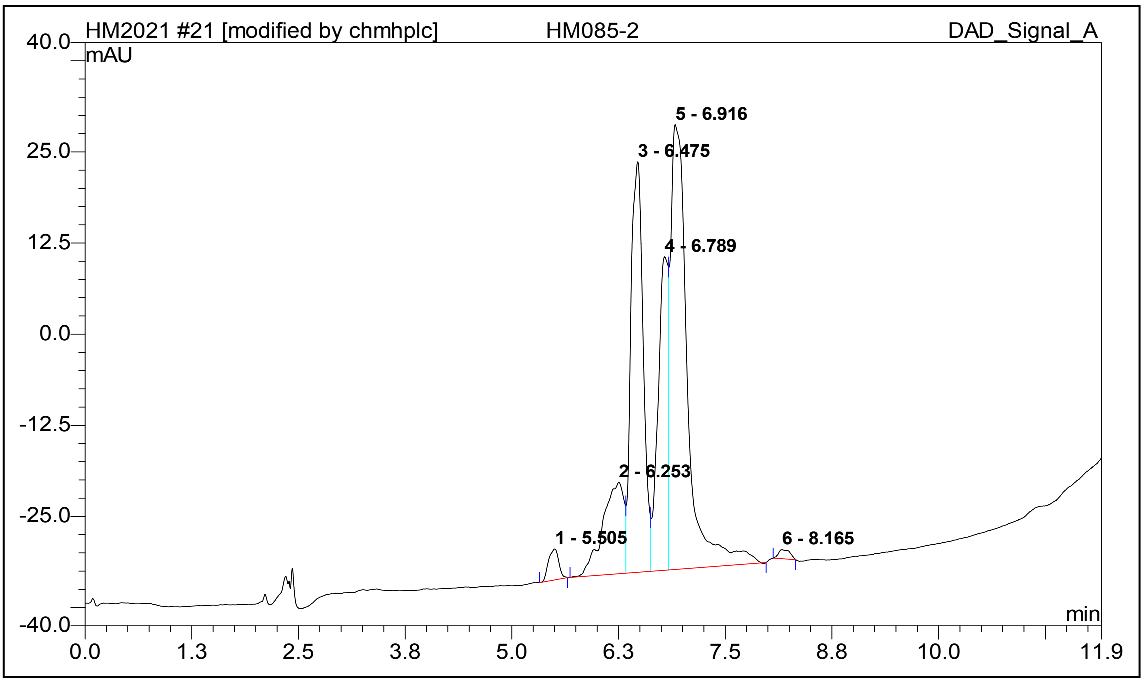

References

1. Dorr, B. M.; Ham, H. O.; An, C.; Chaikof, E. L.; Liu, D. R., Reprogramming the specificity of sortase enzymes. *Proceedings of the National Academy of Sciences* **2014,** *111*, 13343-13348.

2. Zong, Y.; Bice, T. W.; Ton-That, H.; Schneewind, O.; Narayana, S. V. L., Crystal structures of Staphylococcus aureus Sortase A and its substrate complex. *Journal of Biological Chemistry* **2004,** *279*, 31383-31389.

3. Technologies, A., QuikChange II Site-Directed Mutagenesis Kit: Instruction Manual. **2015**.

4. Merck KOD Hot Start DNA Polymerase. (accessed 26/02/19).

5. Williamson, D. J.; Webb, M. E.; Turnbull, W. B., Depsipeptide substrates for sortase-mediated N-terminal protein ligation. *Nature Protocols* **2014,** *9*, 253-262.

6. Schotten, C., Ueber die Oxydation des Piperidins. *Berichte der deutschen chemischen Gesellschaft* **1884,** *17* (2), 2544-2547.

7. Williamson, D. J.; Fascione, M. A.; Webb, M. E.; Turnbull, W. B., Efficient N-terminal labeling of proteins by use of sortase. *Angewandte Chemie - International Edition* **2012,** *51*, 9377-9380.
